# ACE2: Friend or Foe in Post-COVID-19 Neurodegeneration?

**DOI:** 10.3390/ijms262211104

**Published:** 2025-11-17

**Authors:** Svetlana V. Kononova, Natalia V. Bobkova, Rimma A. Poltavtseva, Sergey Leonov, Gennadiy T. Sukhikh

**Affiliations:** 1 Institute of Cell Biophysics, Federal Research Center “Pushchino Scientific Center for Biological Research of the Russian Academy of Sciences”, Pushchino 142290, Russia; nbobkova@mail.ru (N.V.B.); leonov.sv@phystech.edu (S.L.); 2 National Medical Research Center for Obstetrics, Gynecology and Perinatology Named After Academician V.I. Kulakov, Moscow 117997, Russia; rimpol@mail.ru (R.A.P.); gtsukhikh@mail.ru (G.T.S.); 3 Institute of Future Biophysics, Moscow Center for Advanced Studies, Moscow 123592, Russia

**Keywords:** ACE2, multifunctional protein, renin–angiotensin system, COVID-19, Alzheimer’s disease, neurotransmitter systems

## Abstract

Angiotensin-converting enzyme 2 (ACE2) is a key component of the renin–angiotensin system’s counter-regulatory pathway. ACE2 is a multifunctional protein whose location and form determine its catalytic and non-catalytic functions, including amino acid transport, the creation of structural complexes, adhesion, and involvement in signaling pathways. In addition, ACE2 influences neurotransmitter systems in the brain. As the main receptor for SARS-CoV-2, ACE2 has been the subject of increasing research interest. Although ACE2 levels in the brain are low, brain damage from SARS-CoV-2 increases the risk of neurodegenerative diseases. This review aims to clarify an important issue: does the temporary inactivation of ACE2 by the SARS-CoV-2 spike protein play a role in Alzheimer-like neurodegeneration, meaning that the protein may serve as a biomarker or therapeutic target?

## 1. Introduction

The renin–angiotensin system (RAS), once thought to only regulate blood pressure, is now acknowledged for its important multifunctional role in maintaining homeostasis. Angiotensin-converting enzyme 2 (ACE2), identified in 2000, plays a crucial role in the counter-regulatory aspect of the RAS [1,2]. This discovery highlighted the RAS’s role in not just blood pressure and volume regulation but also in regulating vascular resistance, thermogenesis, electrolytes, glucose, immunity, cell growth, fibrosis, oxidative stress, amino acid transport, and gut microbiome. RAS dysfunction may result in diseases like hypertension, cardiovascular and kidney diseases, obesity, metabolic issues, and neurodegeneration [3,4,5,6,7].

In 2003, it was discovered that ACE2 can be used by the SARS-CoV coronavirus as a receptor for cell entry [8]. SARS-CoV-2, which caused the COVID-19 pandemic, also used ACE2 as a receptor. The main focus during the pandemic was to understand the molecular mechanisms of COVID-19, especially the severe respiratory syndrome caused by SARS-CoV-2. However, cells in the stomach, duodenum, rectum, kidneys, heart, hypothalamus, and cerebral cortex exhibit symptoms of a disease affecting multiple systems [9,10]. The targeted damage seems linked to the virus’s preference for ACE2 in those cells [11]. SARS-CoV-2 alters the RAS and its related systems, such as the kinin–kallikrein (KKS) [12,13,14] and neurotransmitter [15,16,17] systems. Many people who recovered from COVID-19 later experienced lasting effects of the disease, called “long COVID-19 syndrome”, “post-acute sequelae of COVID-19 (PASC)”, or “long COVID” [18,19,20]. Some individuals exhibited symptoms resembling Alzheimer’s disease (AD), as there are shared risk factors between severe COVID-19 and AD [18]. It is evident that post-COVID-19 complications may include neurodegenerative diseases like AD, a disease that represents a tough challenge for society in medical, social, and economic terms. Its diverse nature makes it vital to investigate the molecular processes behind its onset in COVID-19 survivors. Although the molecular mechanisms of ACE2 in post-COVID-19 AD are partly understood, some aspects remain unclear. We particularly highlight ACE2’s role in regulating neurotransmitter systems, which has not been extensively discussed. Given the extensive research on the RAS, we have referenced recent reviews and will guide readers to the original works for further information as necessary. While some topics are not directly tied to ACE2 regulation in the brain, we cover them because our understanding of ACE2 and its molecular mechanisms in the brain remains insufficient.

## 2. Brain RAS and ACE2

**Figure 1:**
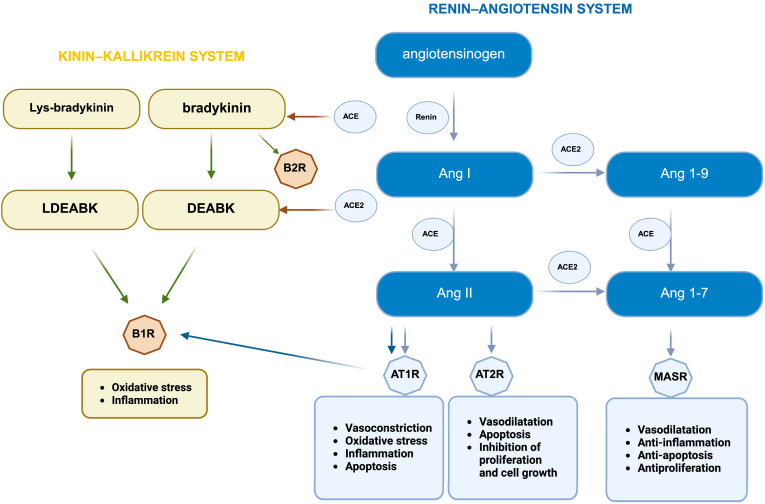
A simplified diagram of the renin–angiotensin system and its interactions with the kallikrein–kinin system. ACE promotes the conversion of angiotensin (Ang) I to Ang II, transforms Ang (1–9) into Ang (1–7), and inactivates bradykinin from the kinin–kallikrein system (KKS). ACE2 facilitates the transformation of Ang I into Ang (1–9) and Ang II into Ang (1–7), as well as breaking down metabolites such as (Des-Arg9)-bradykinin (DEABK) and Lys-(Des-Arg9)-bradykinin (LDEABK). The interaction of Ang (1–7) with the Mas receptor (MASR) can result in vasodilation and antioxidant, antiproliferative, anti-inflammatory, and anti-remodeling effects. Binding of Ang II to angiotensin receptor type 2 (AT2R) can lead to vasodilation, anti-inflammatory and antifibrotic effects, and apoptosis. The binding of Angiotensin II (Ang II) to angiotensin receptor type 1 (AT1R) triggers vasoconstriction, inflammation, fibrosis, and apoptosis, whereas the ACE2/Ang II/bradykinin receptor 1 (B1R) axis contributes to inflammation and oxidative stress [2,12,14,23,25]. The blue arrows represent the reactions occurring in the RAS, while the green arrows illustrate those in the KKS. Additionally, the blue and brown arrows denote the interactions between the RAS and KKS. These involve the Bradykinin receptor 2 (B2R). This figure was designed using BioRender (https://www.biorender.com).

The intricate structure, functions, and regulatory mechanisms of the RAS have been extensively examined in various reviews [1,11,12,21,22]. A concise overview of the key brain components is provided in Figure 1.

ACE is a dual-site dipeptidyl carboxypeptidase. It catalyzes the conversion of angiotensin (Ang) I (1–10) to Ang II (1–8) and inactivates bradykinin from the KKS. Conversely, ACE2 functions as a single-site carboxypeptidase, effectively cleaving a single amino acid from the C-terminus of Ang I and Ang II, leading to the formation of Ang (1–9) and Ang (1–7), respectively. ACE2 is 400 times more efficient at processing Ang II than Ang I. Unlike ACE, ACE2 does not break down bradykinin and is not affected by standard ACE inhibitors. Both peptidases play a crucial role in the hydrolysis of various peptides. Notably, ACE2 can hydrolyze apelin-13, apelin-17, and apelin-36, as well as kinin metabolites like (Des-Arg9)-bradykinin (DEABK) and (Des-Arg10)-kallidin. Additionally, it acts on neurotensin, kinetin, kinetensin, dynorphin A-(1–13), casomorphin-(1–7), ghrelin, and β-amyloid (Aβ) [2,23,24,25].

Ang II engages with two specific receptors, angiotensin receptor type 1 (AT1R) and type 2 (AT2R), with its effects varying based on which receptor is activated (Figure 1). The activation of AT1R triggers oxidative stress, stimulates the hydrolysis of inositol phosphates via phospholipase C, elevates intracellular Ca^2+^ and diacylglycerol levels, enhances sodium retention, suppresses adenylate cyclase activity, and releases metabolites derived from arachidonic acid. This leads to the development of inflammation, fibrosis, and vasoconstriction, which can be aggravated by chronic AT1R activation. Binding of Ang II to AT2R leads to opposite effects: anti-inflammatory, antifibrotic, and vasodilatory. The distribution of these receptors is tissue-specific, and they have been found in the kidneys, lungs, adrenal glands, and brain.

Ang (1–7) primarily binds to specific receptors known as Mas and Mas-related G protein-coupled receptor D (MrgD). At higher concentrations, it may interact with AT1R, altering its effects. The ACE2/ Ang (1–7)/MasR axis serves as a physiological counterbalance to the ACE/Ang II/AT1R axis. This is achieved by counteracting the activated RAS, which reduces Ang II levels while concurrently increasing Ang (1–7) levels. Activation of MAS results in vasodilation and antioxidant, antiproliferative, anti-inflammatory, and anti-remodeling effects. The MrgD receptor interacts with alamandine, a product of ACE2, which originates from angiotensin A and has Ala instead of Asp at the N-terminus, unlike Ang (1–7). ACE2 plays a vital role in the RAS by enhancing the effects of Ang (1–7). It maintains stable blood volume, vascular resistance, and sodium reabsorption, lessening the negative effects of Ang II on AT1R. Ang (1–7) boosts intracellular cAMP levels, activates PKA, and phosphorylates CREB through MasR and MrgD receptors. It also triggers the PI3K-Akt pathway to activate NOS and participates in various other signaling pathways (see reviews [1,11,12,22]).

The discovery of local RAS regulation has led to the identification of “local RASs” in the kidneys, lungs, heart, blood vessels, adrenal cortex, fat, ovaries, testes, skin, and brain, which may have paracrine and autocrine effects [1]. The local brain RAS contains the same major components as the systemic RAS [1,21]. The brain has higher levels of Ang II than blood does, even though Ang II and RAS components generally cannot cross the blood–brain barrier (BBB). This suggests a specific role for the RAS in the brain. Brain Ang II is produced by ACE and enzymes such as chymase and cathepsin G [1]. In the brain, astrocytes, microglia, and neurons generate 90% of angiotensinogen [26], while astrocytes and neurons produce renin [1]. ACE2 activity in the brain is lower than in other body systems but higher than local ACE activity within the brain. The brain’s local RAS is also crucial for cardiovascular health, maintaining water and mineral balance and influencing cognition and behavior through neuroendocrine and stress responses. The localization and activity of ACE2 in brain cells linked to these functions play a crucial role. Notably, ACE2 activity is most pronounced in the hypothalamus, significantly lower in the pituitary gland, and intermediate in the hippocampus. An ACE and ACE2 imbalance is believed to disturb the Ang II/Ang (1–7) ratio and receptor function, potentially linking it to brain issues, hypertension, and neurodegenerative diseases and brain injuries [27].

## 3. ACE2 Expression

ACE2 expression has been observed in various cell types. The tissues containing cell types with >1% proportion of ACE2 expression were discovered within the respiratory system at a rate of 2%, whereas the digestive system showed notably higher levels, especially in the ileum, where it reached 30%. In the genitourinary system, expression levels in the kidneys and bladder reached 4% and 2.4%, respectively. The heart displayed over 7.5% expression in the cardiovascular system, while the central nervous system (CNS) also showed some expression; in the brain, it was significantly lower [11,23].

The varied distribution of ACE2 across different brain regions, combined with the multitude of techniques that have been used to measure it in diverse subjects, organoids, and cell cultures, has led to inconsistent findings regarding the specific cell types that express ACE2 at both the mRNA and protein levels [28,29,30]. The COVID-19 pandemic highlighted the critical importance of studying ACE2 in the brain, as its presence, function, and distribution are directly linked to the virus’s targeting of this organ, causing severe pathological outcomes [9,28,31,32,33,34].

ACE2 is found in various brain areas, such as the choroid plexus, gray matter of the cortex, prefrontal cortex, hippocampus, and olfactory bulb [20]. It was also found in glial cells and neurons [13], with ACE2 expression being observed in both gamma-aminobutyric acid-expressing (GABAergic) and glutamatergic neurons [17]. A brain transcriptome study in humans and mice [29] revealed that the highest level of *ACE2* expression in humans occurs in the substantia nigra (with a positive rate of 3.60%), cervical spinal cord (C-1) (2.52%), hippocampus (2.03%), hypothalamus (1.49%), caudate (1.22%), and anterior cingulate gyrus (1.14%). Significantly lower levels were observed in the amygdala (0.65%), cerebral cortex (0.39%), frontal cortex (0.96%), nucleus accumbens (0.41%), and putamen (0.48%), while there was no expression in the cerebellum. The Human Brain Transcriptome database shows that *ACE2* levels remain stable in the cortex and other brain areas, with slight changes as people age. Analysis of single-cell transcriptome data revealed that only a small percentage of cells expressed *ACE2*, of which ~70% were excitatory neurons, and ~10–20% were interneurons. Astrocytes and oligodendrocytes predominated among the remaining ACE2-positive cell types, while some dopaminergic ACE2-positive neurons were also detected. Overall, *ACE2* expressions in mouse and human brain cells were similar [29]. ACE2 is more prevalent in astrocytes than in neurons in mouse brain slices and is also found in radial glial cells and tanycytes. ACE2 levels in primary astrocytes were significantly higher than in neurons and endothelial cells, according to an in vitro study examining mRNA and protein expression [35].

## 4. ACE2

### 4.1. Human ACE2 Gene, Alternative Splicing and mRNA Transcripts

**Figure 2:**
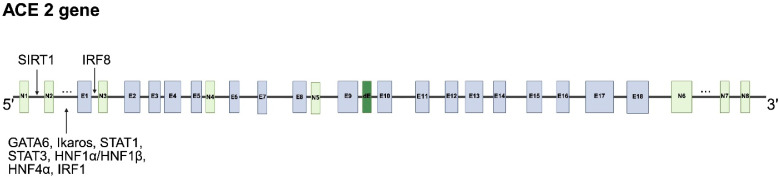
Diagram of *ACE2* gene. The 18 initially identified exons, E1 to E18, are shown in gray, and exon 1 of the truncated ACE2 variant, dE, is highlighted in dark green. Additionally, the predicted exons associated with alternative splicing are depicted in light green [37,38,39,40,41]. Regulation of ACE2 expression at the transcriptional level is controlled by different transcription factors depending on the tissue. The localization of transcription factor binding sites is indicated by arrows [13,39,40,41,42]. This figure was designed using BioRender (https://www.biorender.com).

The human *ACE2* gene is located on chromosome Xp22 and is ~41,036 bp long. The *ACE2* gene exhibits significant genetic variability, as highlighted in [36]. Initially, 18 exons and 17 introns were found [37], but later, exons N1–N5 in the 5′UTR and N6–N8 in the 3′UTR were predicted, suggesting a high potential for alternative splicing [38] (Figure 2).

**Figure 3:**
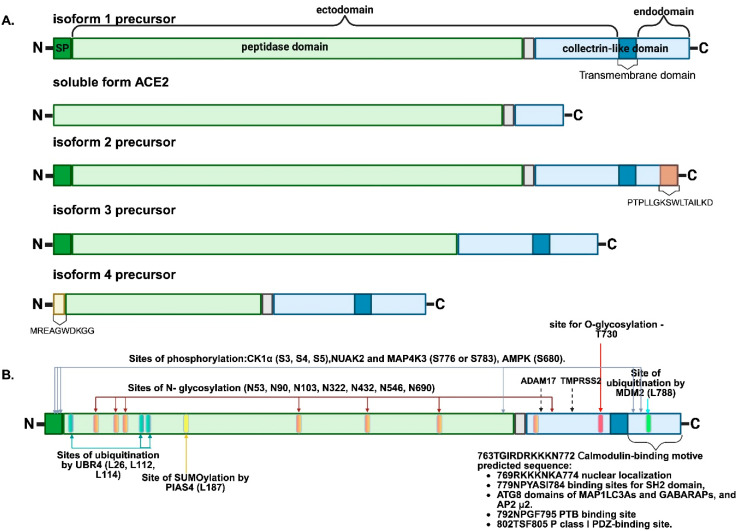
ACE2 protein’s structure. (**A**) A comprehensive diagram of the ACE2 protein’s domain structure, emphasizing its forms and isoforms [39]. (**B**) A schematic representation of the ACE2 protein, indicating sites of sheddase cleavage, post-translational modifications, and its protein interactions. SP—signal peptide. This figure was designed using BioRender (https://www.biorender.com). Panel A was adopted from Stocker et al. (2023) [39] (CC BY4.0).

Alternative splicing produces isoforms that differ in structure and serve various functions in different cell types and organs [38]. There are six distinct variants of mRNA transcripts, which translate into four ACE2 isoforms [39] (Figure 3A).

Full-length ACE2 (805 amino acids (AAs)) is synthesized from two mRNA transcripts of isoform 1 (NP_001358344.1 and NP_068576.1) [39], one of which has a longer 5′-UTR. ACE2 isoform 2 (786 AAs) is encoded by mRNA transcript 3 (NP_001373188.1). It is truncated at the C-terminus [39] and has 100% homology over 770 AA residues with isoform 1 but differs in the last 16 AAs. ACE2 isoform 3 (694 AAs) is also encoded by two mRNA transcripts (NP_001376331.1 and NP_001373189.1). It is 95% similar to isoform 1 but is missing some transmembrane and collectrin-like domains due to splicing and exon loss in the 3′UTR. Isoform 4 (transcript NP_001375381.1) is truncated at the N-terminus due to alternative splicing in the 5′UTR region. This isoform produces a 52 kDa protein composed of 459 AAs, starting with the distinctive sequence MREAGWDKGG and notably lacking carboxypeptidase activity [39,40,41].

ACE2 expression levels can be affected by several factors, such as gender, age, hormones, certain diseases, ACE2 genetic differences, and various medications [11,13,36,43].

### 4.2. Full-Length ACE2

The full-length ACE2 (Figure 3A) comprises a signal peptide (SP) (1–17 AAs), which is cleaved during the co-translational translocation to the endoplasmic reticulum. The molecule includes a significant ectodomain from amino acids 18 to 740, a transmembrane region from 741 to 762, and a short cytoplasmic endodomain from 763 to 805 AAs [44]. Full-length ACE2 is a type I transmembrane protein with a predicted MW of ~92 kDa [39], and glycosylation results in an MW of ~120 kDa [45]. The functional classification identifies two primary subdomains: (1) a zinc metallopeptidase catalytic domain, which shares 42% identity with ACE and features a conserved zinc-binding motif, 374His-Glu-Met-Gly-His378 [2] (located at Exon 9) [37], within its active site; and (2) a collectrin-like domain sharing 48% identity with collectrin [2]. The refined boundaries of the metallopeptidase domain lie between 19 and 607 AAs [46], and the collectrin-like domain from 619 to 805 AAs [46,47].

The ectodomain of ACE2 allows it to hydrolyze circulating peptides. The optimal pH for ACE2 is around 6.5. Its catalytic activity remains stable between pH 7 and 9 but drops at pH 5 [24]. ACE2 has one chloride site CL1 and its catalytic activity is regulated by chloride ions (Cl^−^). Mutational analysis and crystal structure studies of the ACE2 CL1 site have identified Arg169, Trp477, and Lys481 as key amino acids [48,49].

The Arg514 residue is important for selecting substrates, working with Tyr510, Phe504, and Thr347 to alter the enzyme’s active site environment. These residues create conditions that are conducive to Ang II hydrolysis while rendering Ang I hydrolysis less favorable [48,49]. Glu273 in ACE2, rather than Arg273 in ACE, determines the difference in substrate specificity between ACE and ACE2 [50]. However, the function of ACE2 is not limited to an enzymatic one, as will be shown below. The ACE2 protein can also perform various non-catalytic functions.

Regulation of the functional activity of ACE2 is carried out at the level of transcription, post-transcriptional regulation and post-translational modifications (discussed in detail in reviews [36,42]).

### 4.3. Regulation at the Transcriptional Level

The proximal or distal part of the promoter of the human and mouse *ACE2* genes is used, depending on the tissue [13]. Transcription factors that have been identified for the *ACE2* promoter of humans and mice include GATA6, Ikaros, STAT1, STAT3, HNF1α, HNF1β, HNF4α, and SIRT1 [42] (Figure 2). The *ACE2* gene’s distal promoter region contains binding motifs for MYBL2, USF1, TAED4, SP1, CEBP, MAFF, and GATA3. Nucleosome-free regions are found in alveolar cell types 1 and 2, as well as secretory, multiciliated, ionocyte, and neuroendocrine cells, and are occupied by IRF1, STAT1/2, FOXA1, and FOXD2 [13]. The binding of STAT1, STAT3, IRF8, and IRF1 to the *ACE2* promoter shows that *ACE2* is an interferon-inducible gene (ISG) [13]. Notably, a functional p53 binding site was found in the *ACE2* gene promoter in pig PKDNF cells. The tissue specificity and gender pattern of ACE2 expression are similar in humans and pigs. TP53 knockout altered ACE2 protein expression in a cell-type- and gender-specific manner [51]. Various factors, including DYRK1A, interferons (IFNs), SMAD4, EP300, PIAS1, and BAMB, have been shown to enhance the transcription of *ACE2*, whereas Brg1-FoxM1, ERRα [43], and Nrf2 [52] suppress its transcription. Sirtuin 1 (SIRT1) is an enzyme that removes acetyl groups from histones and acts as a transcription factor, playing a role in cellular aging and energy balance [42,53]. The binding of SIRT1 to the *ACE2* promoter induces *ACE2* transcription. When energy stress occurs (high AMP-to-ATP ratio), AMPK (AMP activated protein kinase) is activated, leading to increased *ACE2* transcription through the SIRT/AMPK pathway. SIRT1 stimulates phosphorylation and activation of AMPK, which can reciprocally activate SIRT1 [42].

Epigenetic changes like histone acetylation and methylation help cells adjust their gene expression based on environmental cues. Epigenetic regulation of *ACE2* gene transcription is mediated by K27 acetylation or K4 methylation of histone H3. Monomethylated histone 3 lysine 4 (H3K4me1), trimethylated histone 3 lysine 4 (H3K4me3), and monoacetylated histone 3 lysine 27 (H3K27ac) interact with the *ACE2* locus within human lung tissue [42]. Histone methyltransferase EZH2 (enhancer of zeste homolog 2) catalyzes di- and trimethylation of Lys H3 residues. Trimethylation of Lys 27 on histone 3 (H3K27me3) prevents H3K27ac from binding to the *ACE2* promoter, which inhibits *ACE2* gene transcription in mammalian cells [54]. H3K27ac binds to the *ACE2* promoter, epigenetically enhancing *ACE2* transcription [42]. Lysine demethylase 5B (KDM5B) removes methyl groups from H3K4me3 and reduces the expression of miR-125a and the miR-200 family, indirectly affecting *ACE2* transcript levels [13].

*ACE2* expression can be regulated by epigenetic factors, including smoking and sex hormones, during fetal development and in diseases like autoimmune, cancer, and metabolic diseases [36,44]. It was reported that the *ACE2* gene methylation rate was lowest in the lungs and highest in neurons [44]. Age-related epigenetic changes in the brain lead to decreased ACE2 function and neuronal death [18].

### 4.4. Regulation of ACE2 mRNA Expression by Non-Coding RNAs

Recently, 1954 microRNAs involved in the *ACE2* expression regulatory network have been identified. Certain entities can directly control *ACE2* mRNA expression by targeting the 3′UTR, leading to degradation or translation disruption; they can also influence *ACE2* expression indirectly [36,44]. Some of these have been the main focus of research on various pathologies, including AD and COVID-19.

hsa-miR-125a, hsa-miR-200, hsa-miR-125b-5p, hsa-miR-141, hsa-miR-9-5p, and hsa-miR-218-5p can target the 3′-UTR of *ACE2* to suppress its expression [55]. miR-125b is linked to neuropathological processes like AD dementia, yet it typically functions as a tumor suppressor in cancer [56]. Of note, miR-125b is one of the miRNAs that are associated with cellular stress and AD pathology [18]. Additionally, miR-125b reduces *ACE2* mRNA stability in high-glucose conditions and causes more reactive oxygen species (ROS) and increased cell death in HK-2 and HEK-293T renal tubular epithelial cells [36,42]. Hsa-miR-18 and hsa-miR-125b significantly contribute to acute kidney injury in SARS-CoV-2 patients by interacting with the 3′-UTR of *ACE2* [57]. In senescent cells, ACE2 and miR-18a are downregulated. Exosomal ACE2 from primed endothelial progenitor cells with miR-18a helps protect endothelial cells against hypoxia/reoxygenation injury by suppressing the Nox2/ROS pathway [44].

miR-9-5p is generally downregulated in AD patients, although some studies show that it is upregulated in the hippocampus and temporal lobe neocortex. MiR-21-5p is upregulated in AD and inhibits Aβ-induced cell apoptosis [58]. Downregulation of miR-98 and miR-223 resulted in decreased ACE2 expression in bronchial stem cells during SARS-CoV-2 infection [44]. Both miRs are involved in the pathogenesis of AD [59,60].

The upregulation of miR-200c-3p and the condition of systemic arterial hypertension stand out as independent determinants of severe COVID-19 [61]. On the one hand, the expression of miR-200c is upregulated in response to oxidative stress; on the other hand, this miR reduces Aβ secretion in the AD model [62]. Binding of miR-200c-3p and miR-421-5p to the 3′-UTR region of *ACE2* resulted in decreased *ACE2* mRNA stability [13,36,42,61,63]. Moreover, cell culture studies showed that not only *ACE2* mRNA but also ACE2 protein expression was suppressed [36].

Ang II-induced miR-21 is implicated in lung fibrosis by decreasing Spry1 in lung fibroblasts, which leads to inflammation and more collagen production. PYrin domain-containing protein 3 (PYD) inflammasome (NLRP3) is an inflammatory signaling pathway that is often stimulated by ROS and Nuclear factor-κB (NF-κB) signaling [26]. It plays a vital role in AD progression, especially when activated by aggregated Aβ [58]. ACE2/Ang (1–7) suppressed Ang II-induced ERK (Extracellular signal-regulated kinase) phosphorylation, NF-κB nuclear translocation, *Spry1* gene degradation, and Ang II-induced NLRP3 inflammasome activation. Neither ACE2 nor Ang (1–7) affected the function or downstream molecular signaling of miR-21, but they did affect its expression. miR-21 mediates ACE2/Ang (1–7) inhibition in Ang II-induced NLRP3 inflammasome activation [64].

AT1R-regulated miR-483-3p can inhibit the expression of four crucial RAS components in vascular smooth muscle cells (VSMCs): ACE2, angiotensinogen (AGT), ACE, and AT2R [36,42]. However, in HEK-293T cells with constitutive miR-483-3p expression, *ACE2* mRNA levels were not changed [36]. Whether miR-483-3p regulates ACE2 expression in the AD brain is unknown.

In cell cultures (HEK293T, Huh7, cardiac myofibroblasts), miR-421 overexpression reduced ACE2 protein levels by binding to the *ACE2* 3′-UTR [42]. MiR-421 plays an important role in neuronal diseases [65].

Various physiological conditions can affect *ACE2* expression through a range of miRNAs. Increased miR-let-7b expression in hypoxia, driven by HIF-1α, lowers *ACE2* levels in pulmonary artery smooth muscle cells (PASMCs) from Sprague-Dawley rats and C57Bl/6 mice [36]. Hypoxia significantly increases the risk of developing neurodegenerative diseases, in particular AD, through dysregulation of the HIF-1α pathway [17]. HIF-1α affects *ACE2* mRNA and proteins based on the stage of hypoxia and the specific cell type. In the early phase of hypoxia, HIF-1α raises *ACE2* mRNA and protein levels in PASMCs, but these are reduced later [66]. In accordance, patients with pulmonary fibrosis showed lower *ACE2* expression when experiencing hypoxia [36]. The lncRNA ALT1 was found to interact with ACE2 protein in human umbilical vein endothelial cells. *ALT1* knockdown combined with contact inhibition led to lower ACE2 and higher HIF-1α levels. In contrast, enhancing ACE2 expression subsequently lowered HIF-1α levels, pointing to a feedback mechanism. Targeting either ALT1 or ACE2 substantially decreased cyclin D1 levels, driven by increased ubiquitination and degradation processes linked to HIF-1α and the von Hippel–Lindau protein (pVHL) [67].

Infection with SARS-CoV-2 leads to elevated miR-1246 and miR-1290 levels in lung epithelial cells [68]. Prior research involving pulmonary microvascular endothelial cells (PMVECs) [69] and Calu-3 cells [68] has shown that miR-1246 inhibits *ACE2* expression by interacting with the 3′UTR. miR-1246 was upregulated in C57Bl/6 mouse PMVECs in response to LPS treatment and decreased ACE2 expression. In small airway epithelial cells, smokers had lower miR-1246 expression than non-smokers, whereas *ACE2* mRNA was increased [70]. miR-1246 significantly influences apoptosis in pulmonary endothelial cells through targeting ACE2, thereby affecting acute lung injury and acute respiratory distress syndrome [69]. It is worth noting that the impact of miR-1246 on ACE2 is not a default process and does not always degrade *ACE2* mRNA [68]. Both hsa-miR-1246 and miR-1290 are considered non-invasive biomarkers of AD [71,72]

Recent findings highlight a range of non-canonical mechanisms by which miRNA can regulate target mRNA [73].

### 4.5. Alternative Regulation of ACE2 mRNA Expression

Besides miRNAs, *ACE2* mRNA levels can be influenced by the peptide apelin (APLN), interleukin 1β (IL-1β), and chitinase 3-like-1 (CHI3L1). APLN interacts with its receptor APLNR (APJ), which is similar to the antagonist AT1R. This interaction increases *ACE2* transcription in heart cells and improves cardiac function independently of AT1R signaling [42]. APJ physically interacts with AT1R, placing it in a low-affinity state and reducing Ang II binding and signaling. The −252 to −202 bp promoter region in Vero E6 cells contains an HNF1-β binding site, which is crucial for APLN to activate *ACE2* transcription. However, APLN activated the minimal *ACE2* promoter from −202 to +103 bp in HEK293 cells, questioning HNF1-β’s role in transcription [74]. Notably, apelin-13 upregulated the brain neurotrophic factor (BDNF)/tropomyosin receptor kinase (Trk) pathway against cognitive deficit by attenuating inflammation in a streptozotocin-induced AD rat model [75].

CHI3L1, belonging to glycoside hydrolase family 18, is known as a biomarker of neuroinflammation and AD diagnosis [42,76]. Circulating CH3IL1 levels were elevated in elderly patients with severe COVID-19 and comorbidities. Recombinant CH3IL1 increases mRNA levels of *ACE2*, *TMPRSS2* (Transmembrane protease, serine 2), and *CTSL* (Cathepsin L) in human lung epithelial cells (Calu-3, A549, HSAEC) and lung fibroblasts [42,77].

Inflammation can affect *ACE2* expression significantly, since various factors—including NF-κB, IFN-γ, tumor necrosis factor alfa (TNF-α), IL-1β, IL-4, and transforming growth factor beta (TGF-β)—can regulate it. Inflammasomes are a source of IL-1β [36]. IL-1β treatment of osteosarcoma U2OS and MNNG-HOS cells raised *ACE2* and *MAS* mRNA levels, inhibiting their growth and migration [42]. In cardiac fibroblasts, the AngII/AT1R axis also stimulates NLRP3 inflammasome expression through Ca^2+^ release [26]. TNF-α reduces *ACE2* mRNA levels in the ileum of Crohn’s disease patients but increases them in the colon of ulcerative colitis patients [42]. However, in the AD brain, TNF-α likely regulates ACE2 levels indirectly through the sheddase ADAM17 (discussed in Section 4.6 ACE2 Processing by Proteases).

Hypertensive patients have increased ACE and decreased ACE2 levels in cardiopathy and nephropathy. Ang II can boost ACE levels while lowering *ACE2* mRNA and protein in hypertension through the AT1R pathway that activates ERK/p38 MAP kinase (mitogen-activated protein kinase) [42,78]. Ang II is involved in the development of pulmonary fibrosis via the Smad/TGF signaling cascade. After Ang II binds to AT1R, activation of the MAPK/ERK pathway leads to phosphorylation of Smad2 and Smad3. Phosphorylated Smad2 and Smad3 then bind with Smad4, moving into the nucleus to initiate the transcription of TGF-β, fibronectin, and procollagen I genes [79]. Ang II increased AT1R levels and phosphorylation of ERK1/2 and STAT3 while inhibiting ACE2 mRNA and protein levels. In VSMCs, Ang II treatment increased SOCS3 expression and activated JAK2, STAT3, and ERK1/2, leading to increased superoxide production and cell division. ACE2 mitigates oxidative stress and VSMC growth by suppressing AT1R and the Ang II-triggered JAK2/STAT3/SOCS3 and profilin-1/MAPK pathways [80,81]. Stimulation of renal cells by TGF-β causes upregulation of Adam10, 12, 17, and 19 [82]. In hyperglycemia, increased TGF-β in kidneys causes diabetic nephropathy and lowers ACE2 mRNA and protein levels in glomerular and tubular cells. TGF-β treatment inhibits *ACE2* gene expression in the proximal tubules of NRK-52E rat kidneys [42]. Blocking TGF-β-SMAD2/3 signaling ameliorates AD pathology [83].

17β-estradiol, a key estrogen, lowers ACE2 expression and activity in the kidneys and human airway epithelial cells, regardless of gender. However, in human atrial tissue, estrogen treatment increases ACE2 mRNA and protein levels via the estrogen receptor α-activated pathway. It is suggested that the effect of estrogen on ACE2 levels may be tissue-specific [42,44].

### 4.6. Post-Translational Regulation of ACE2 Expression

ACE2 protein can undergo various post-translational modifications that regulate its levels and activity (Figure 3B). For example, ACE2 expression levels in endothelial cells are regulated through a phosphorylation/ubiquitination mechanism involving AMPK and the E3 ligase MDM2 (mouse double min 2). AMPK phosphorylation of Ser680 on ACE2 boosts protein levels by stopping ubiquitination and proteasomal degradation [42]. In a mouse model of LPS-induced acute lung injury, ACE2 enhanced AMPK phosphorylation, inhibiting mTOR function via mTORC1 [84]. In AD and mice with subarachnoid hemorrhage in the cerebral cortex, ACE2 restored autophagic flow by activating the PI3K/AKT pathway, reducing neuron damage. PI3K/AKT is one of the main pathways regulating autophagy by stimulating mTOR [85]. In hypertensive rat myocardial cells, overexpression of SIRT6 deacetylase results in AMPK activation and ACE2 induction. Phosphorylation and stabilization of ACE2 by AMPK may help avoid lung and heart damage from pulmonary hypertension and heart failure [42]. AMPK phosphorylation at Ser680 prevents ACE2 from being ubiquitinated by the E3 ligase MDM2 at Lys788, stopping its degradation. Under hypoxia, MDM2 attaches ubiquitin to ACE2 at Lys788, leading to dysfunction in endothelial cells and a lower ACE2-to-ACE ratio in the RAS [42]. In addition to MDM2, other E3 ligases, like Neural precursor cell expressed developmentally downregulated gene 4-like (NEDD4L) and Mindbomb E3 ubiquitin protein ligase 1 (MIB1), are strong candidates for the ubiquitination of ACE2 [86]. Overexpression of Ang II and AT1R enhances ACE2 ubiquitination in human HEK293T cells. In male mice treated with Ang II, NEDD4L and ACE2 protein levels were inversely correlated in the brain, heart, and kidney. Ang II decreases ACE2 protein through NEDD4L-mediated ubiquitination, causing hypertension, while ACE2 regulation in female mice may not be linked to Nedd4-2 [42]. S-phase kinase-associated protein 2 (Skp2), an E3 ubiquitin ligase, influences the cell cycle by degrading tumor suppressor genes like p21, p27, and p57, as well as causing the ubiquitination and degradation of ACE2 in lung epithelial cells [42]. Knockdown of *SKP2* worsened cognitive impairment, inhibited neuronal regeneration, and promoted Aβ deposition in a mouse model of AD [87].

Ubiquitin carboxyl-terminal hydrolase L1 (UCHL1) is highly expressed in the central nervous system, and its dysfunction leads to the development of neurodegenerative diseases, including AD [88]. It stabilizes ACE2 protein in Calu-3 and Bease2B cells by stopping the degradation caused by the SARS-CoV-2 S protein, but the level of *ACE2* mRNA is not changed [42].

There are a number of other ACE2-phosphorylating kinases and enzymes that affect its ubiquitination, such as casein kinase 1 α (CK1α) [42], NUAK family kinase 2 (NUAK2) [42], MAP4K3 (GLK) [89], E3 ligase UBR4 [42] and ubiquitin-specific peptidase 50 (USP50) [42], whose expression was increased in COVID-19 patients [42], but their association with AD has not been shown.

SUMOylation of proteins results in changes in protein function, stability, or localization, often by modulating interactions [90]. Protein ubiquitin-like modifiers (SUMOs) attach to target proteins through an E1 activating enzyme, an E2 conjugating enzyme, and an E3 ligase. The E3 ligase protein inhibitor of activated STAT4 (PIAS4) has been shown to induce SUMO3 SUMOylation of ACE2 at Lys187. This leads to the stabilization of the ACE2 protein [42]; however, PIAS4 expression in hippocampal and cortical neurons was not detected [91].

Yet another post-translational modification is methylation of Arg671 in ACE2 by arginine methyltransferase 5 (PRMT5), which leads to the creation of symmetric dimethylarginines, significantly facilitated by the interaction between the SARS-CoV-2 receptor-binding domain (RBD) and ACE2, both in vitro and in vivo [42]. PRMT5-mediated modification of Arg671 probably promotes Asn322 N-glycosylation in ACE2 [92]. In summary, it is important to note that glycosylation stands out as one of the major post-translational modifications of ACE2, typically affecting either the conformation (discussed below) or cleavage sites that are recognized by diverse proteases. Human ACE2 (hACE2) has seven sites for N-glycosylation (Asn at positions 53, 90, 103, 322, 432, 546, and 690) and one for O-glycosylation (Thr730). Aside from Asn432 and Asn690, which exhibited 26.8% and 1.1% reductions in glycosylation, all other sites were fully glycosylated. Glycans were predominantly of the complex type, of which ~60% were biantennary, although triantennary and quaternary structures were also present. More than 85% of the structures were fucosylated, and approximately half were sialylated [93].

### 4.7. ACE2 Processing by Proteases

Full-length ACE2 [12] is a membrane protein (mACE2). Its soluble form (sACE2) can be released into the bloodstream or urine, which is facilitated by specific enzymes known as sheddases—namely, ADAM17 and ADAM10—which both belong to the membrane protease family, characterized by their disintegrin and metalloproteinase (ADAM) domain [46,94]. Different studies have demonstrated discrepancies in the location of the ADAM17 cleavage site in ACE2. An earlier study identified localization from 716 to 741 AAs [44] and noted Leu584’s potential involvement in the ADAM17 recognition motif. Further analysis indicated that the cleavage site is between Arg708 and Ser709. Another study identified Arg652, Lys657, and Lys659 as essential components of the ADAM17 cleavage site [95].

In the absence of disease, ADAM17 and ADAM10 release low levels of sACE2 in specific tissues [25,46]. This is thought to be a potentially important mechanism for regulating local mACE2 activity and Ang (1–7) concentrations [24]. Various studies [25,46] have deemed each sheddase to be essential, but their functions likely depend on specific contexts. Hence, the EGFR/ADAM17 pathway can be anti-inflammatory or pro-inflammatory based on the stimulus, substrate, and type of cell involved [43]. The transport process of ADAM17 from the endoplasmic reticulum to the Golgi apparatus, including its subsequent modification and interactions with various substrates, is governed by iRhom1 and 2, which belong to a conserved superfamily of proteins that interact with membrane proteins and direct them to diverse cellular pathways [82,96]. In the brain, ADAM17 maturation is regulated by iRhom1, except in microglia, where this function is performed by iRhom2 [82]. In inflammatory conditions, excessive proinflammatory iRhom2 expression amplified the release of ACE2 through ADAM17 [46].

ADAM17, also referred to as TNF-α-converting enzyme (TACE), facilitates the release of soluble active TNF-α [82]. ADAM17 activity can be triggered by factors such as apoptosis, Ang II, D-glucose, IL-1β, Ca^2+^ ionophores, fibroblast growth factor 7 (FGF7), protein kinase C (PKC) activators, and purine receptor 2 (P2) agonists during pathogen infection through toll-like receptors (TLRs). It is dependent not only on the stimulus but also on the cell type [25,43,82].

Phosphorylation of ADAM17’s cytoplasmic domain by kinases like PKC, PLK2, or MAPK boosts its activity [96]. The resulting rise in sACE2 levels, along with a local reduction in mACE2, impairs the conversion of Ang II into Ang (1–7). Consequently, this shift favors Ang II, which activates ADAM-17, creating a self-reinforcing feedback loop. This process facilitates the continuous shedding of mACE2, which in turn drives the overactivation of RAS and leads to increased inflammation [96]. Plasma sACE2 levels increase with age, with higher levels in men than in women [43]. The production of a catalytically active form of sACE2 by ADAM17 is often seen in pathological states [96], including diabetes [5] or neurogenic hypertension in mice, as well as hypertension in humans [2,82]. Excessive RAS activity in hypertensive patients leads to the development and progression of Alzheimer’s disease by activating neuronal AT1R, resulting in oxidative stress and neuroinflammation. AT1R promotes the accumulation of Aβ and neurofibrillary tangles (NFTs) [26]. Higher Ang II levels in Neuro-2A cells result in decreased ACE2 protein expression, mediated by AT1R. At low levels of Ang II, ACE2 engages in a relationship with AT1R. However, as Ang II levels rise, this ACE2-AT1R complex shifts into the endosomal pathway, leading to its dissociation. Thereafter, ACE2 is ubiquitinated and degraded in lysosomes, while AT1R is brought back to the membrane through endosomal transport [44]. The COVID-19 pandemic led to the exploration of recombinant sACE2 (rsACE2) as a potential decoy to prevent SARS-CoV-2 infection [97,98]. However, research on the HK-2 human kidney cell line shows that using rsACE2 at physiological concentrations can increase SARS-CoV-2 infection. This enhancement happens because of the interaction between rsACE2 and the virus’ S proteins, causing endocytosis through either AT1R or the vasopressin receptor VPR1B. The S protein further facilitates the formation of the rsACE2–S protein–vasopressin complex [99]. At the same time, new findings indicate that the decoy receptor (ACE2-Fc) may effectively combat the rapidly mutating SARS-CoV-2 and address immune evasion [100].

Whether the enzymatic activity of sACE2 has any pathophysiological significance remains an open question. sACE2 could potentially interact with other cells through integrins, activating signals related to cell growth and survival [45]. mACE2 can act as a ligand for cell adhesion and binds integrin β_1_ (ITGB1) and integrin α5 (ITGA5) in vitro. sACE2 significantly decreased phosphorylated focal adhesion kinase (pFAK) levels in Huh7 cells and primary myofibroblasts but increased Akt expression levels. Akt signaling was not transduced to its downstream effector NF-κB. Despite having catalytic activity, sACE2 mediates signaling independently of its product Ang (1–7), suggesting that its signaling function does not rely on this catalytic role. It was suggested that sACE2, through its integrin binding properties, may play a regulatory role in cell–cell interactions and support a novel mechanism of integrin activation through ACE2 shedding [45].

Research indicates that sACE2 may play a role in tissues exhibiting low levels of ACE2 expression [46]. The remaining intracellular part of ACE2 after ADAM17 proteolysis can be targeted for regulated intramembrane proteolysis (RIP) and is prone to degradation. Furthermore, when ACE2 cleavage is constitutively regulated by ADAM17, its degradation takes place through the proteasome. When Ang II levels rise and activate ADAM17, AT1R is essential for ACE2 internalization. This leads to the ubiquitination of the ACE2 endodomain by MDM2 and Skp2, which is then targeted for degradation within the lysosomes, while AT1R is recycled back to the membrane [25]. In particular, the latter mechanism was observed in ACE2-GFP-transfected Neuro-2A cells [101].

Activated MasR connects various signaling pathways like MAPK (p38, ERK1/2, JNK), NF-κB, AKT, and SHP2/ROS/HIF1α, influencing ACE2’s role in health and disease [102]. Research on SARS-CoV-2’s entry into Caco-2 cells reveals that EGFR assists ACE2 in binding to the Spike protein’s RBD due to the presence of ADAM17 and TMPRSS2. AT1R, ACE2, and EGFR likely form dynamic complexes on cell surfaces, influenced by ligands or physiological conditions. In the absence of Ang-II, ACE2 in the AT1R-ACE2 complex does not internalize or undergo ubiquitination. However, when Ang-II and EGF are present, EGFR is activated in the AT1R-EGFR complex [103,104]. Therefore, it appears that catalytically active mACE2 suppresses EGFR activation [103]. Binding of recombinant Spike RBD or pseudovirus Spike protein to ACE2 caused EGFR phosphorylation, temporarily activated the EGFR–CRAF–MEK1/2–ERK1/2 pathway, and led to the movement of ACE2 and EGFR to early endosomes. This activation was specifically Spike protein-mediated and did not coincide with SARS-CoV-2 replication in cells. ERK1/2 and CRAF are essential for MAPK signaling, which controls cell growth, differentiation, survival, and migration [103].

In addition to ADAM17 and ADAM10, ACE2 proteolysis can be performed by TMPRSS2, human respiratory trypsin (HAT), and hepsin. For ACE2 proteolysis, 697 to 716 AAs are critical for TMPRSS2 and HAT [105]. ADAM17 competes with TMPRSS2 for ACE2 processing. The ACE2 ectodomain that is processed by TMPRSS2 has no catalytic activity [105]. A C-terminal fragment of ACE2 was reported to be detected in a case of TMPRSS2-mediated ACE2 cleavage. It is unclear if the undegraded cytosolic fragment of ACE2 can move to the nucleus, but a potential nuclear localization sequence (769RKKKNKA774) has been identified in its endodomain. It is also unknown whether it can have any physiological function through interactions with other cytosolic proteins [25]. The ACE2 endodomain contains a calmodulin-binding motif (763–772 AAs) [24]. The full-length ACE2 binds to calmodulin, which protects it from being broken down by ADAM17 and TMPRSS2, indicating that calmodulin helps stabilize the active form of mACE2 [106]. The binding of ACE2 to Ca^2+^ might cause changes that impact how easily proteases can access the cleavage site. It has been reported that an increase in Ca^2+^ levels following ionomycin treatment induces ACE2 cleavage by ADAM10 and release of the ectodomain [25].

### 4.8. ACE2: A Chaperone for the Membrane Trafficking of Amino Acid Transporters

The collectrin-like domain of ACE2 is thought to have formed from the fusion of the *Ace* and collectrin genes in early evolution [50]. The ACE2 homodimer can create heterodimers with the sodium-dependent amino acid transporters B^0^AT1, B^0^AT3, and SIT1, which are encoded by the *SLC6A19*, *SLC6A18*, and *SLC6A20* genes [107,108]. The collectrin-like domain enables ACE2 to act as a chaperone for transporter proteins, aiding in their transport, insertion, and stable function in the membrane. A genome-wide study identified *SLC6A20*, together with *LZTFL1*, *CCR9*, *FYCO1*, *CXCR6*, and *XCR1*, as a notable gene tied to severe COVID-19 risk [109].

Cryoelectron microscopy was used to determine the full-length hACE2 structure in the presence of B^0^AT1, which stabilized it [110]. The ACE2 dimer can exist in “closed” and “open” conformations, which are found in a 3:1 ratio, as well as in an intermediate conformation [23]. ACE2 forms dimers at two sites: the first site is in the peptidase domain, which triggers changes from a “closed” to “open” conformation [111], while the second site (697–716 AAs) has stronger polar interactions in the collectrin-like domain than the first [110]. The extracellular parts of the proteins are stabilized by the so-called neck domains (616–726 AAs). In the “closed” conformation, the dimer’s catalytic domains face and interact with each other, like the neck domains. They can perform hinge-like movements when changing from one conformation to another. The “open” conformation occurs when the dimer interaction between the catalytic domains breaks, yet the dimer remains stable because of neck domain interactions [23]. N-glycans at Asn432 regulate the dimerization process, while the Asn690 site is crucial for protein folding and trafficking [112]. O-glycosylated Thr730 is likely to be involved not only in dimerization and ACE2 presentation on the cell surface but also in the regulation of ectodomain shedding [93]. The glycan at Asn432 is believed to stabilize the ACE2 dimer’s transition between its “open” and “closed” forms by modifying electrostatic interactions, as it stays “closed” without the N-glycan [112]. The N-glycan chain at Asn690 of ACE2 is rigid and stable in the “open” conformation, functioning as a latch on the peptidase domain. In the “closed” conformation, the protein–glycan contact points are fewer [113]. The transition between these conformations can form an “intermediate” conformation, which can also be stabilized. Behind the active site, there is a claw-like surface where the RBD of SARS-CoV Spike proteins can bind [23]. In the RBD-ACE2-B^0^AT1 complex, ACE2 was first found in the “closed” state [110], but later studies showed that it can also be “partially open” with the SARS-CoV or SARS-CoV-2 RBD [114]. The second ACE2 site is believed to play a role in regulating B^0^AT1 transport [50]. It interacts with certain residues (616–726 AAs) and a transmembrane helix of ACE2 when forming a heterodimer [110]. In the ACE2-B^0^AT1 heterodimer, ACE2 is likely protected from TMPRSS2 cleavage, because B^0^AT1 blocks the ACE2 cleavage site (697–716 AAs) [110].

The neutral amino acid transporter B^0^AT1 has a broad substrate selectivity: Met = Leu = Ile = Val > Gln = Asn = Phe = Cys = Ala > Ser = Gly = Tyr = Thr = His = Pro > Trp > Lys [115]. It was also reported that it can transport Arg, along with Lys [116]. The ACE2-B^0^AT1 complex in the small intestine regulates the entry of dietary Trp into cells [117], which, through activation of the mTOR pathway, increases the mRNA of the antimicrobial peptide Defa1, controls the innate immune response, and modulates intestinal microbiota [118]. ACE2 and collectrin form specific heterodimers with B^0^AT1 in different tissues: ACE2 in the small intestine and collectrin in the kidney’s apical brush border epithelium [119]. ACE2 is found in enterocytes of the duodenum, jejunum, and ileum, but not in those of the colon and stomach [13]. The highest expression of *ACE2* mRNA was found in ileal enterocytes, with a decrease being noted in the transverse colon; meanwhile, *TMPRSS2* expression displayed an opposing trend [120]. *SLC6A19* was most expressed in the ileum, less in the jejunum, and absent in the cecum and colon [121]. These findings suggest that ACE2 exists as a homodimer in both the transverse colon and the kidneys, serving an exclusively catalytic role. While the expression of *Slc6a19* in the brain has not been documented before, recent findings indicate that both *Slc6a19* mRNA and *Ace2* mRNA, along with its protein, are present at low levels in the ependymal cells of the mouse brain. Additionally, *Cltrn* mRNA expression has been observed in the choroid plexus of the brain [122]. This may indicate the functioning of the ACE2-B^0^AT1 transporter in ependymal cells of the brain. *SLC6A19* expression might be influenced by hypoxia through HIF1, indicating its possible role in cerebral ischemia and hypoxia [122].

The substrate selectivity of the other two transporters that form heterodimers with ACE2 is narrower. SIT1 transports Pro, Gly, hydroxyproline, betaine, N-methylaminoisobutyric acid, and pipecolic acid. Its active site is believed to share features with the GABA transporter GAT1 [108,123]. B^0^AT3 is more effective in transporting Ala, Met, Val, and Ile than it is Gly, Ser, and Leu [124]. *SLC6A20* is expressed on the luminal membrane of enterocytes in the small intestine and renal proximal tubule cells [125]. SIT1 has been found in brain cells, particularly in neurons that use Pro to synthesize Glu [108]. In the intestine, the ACE2-B^0^AT1 and ACE2-SIT1 clusters, along with the Na^+^/glucose co-transporter SGLT1 (from *Slc5a1*), create a metabolic and signaling hub. This supramolecular structure enables Na^+^ and organic osmolytes to enter, triggering water flow through the aquaporins AQP1 and AQP5. Na^+^-dependent glucose uptake contributes to 40% of cellular glucose intake, while insulin secretion, regulated by the pancreatic ACE2-B^0^AT1 complex, enhances glucose entry through glucose transporter 4 (GLUT4) [106]. Balanced levels of organic osmolytes, ions, and water help maintain physiological cellular volume [106].

In complexes with ACE2, B^0^AT1 or SIT1 remains stable, regardless of whether the substrate is bound or not. The binding pattern of AA substrates differs among the complexes, depending on the AA type. ACE2 may play a direct role in regulating the transport activity of ACE2-B^0^AT1 and ACE2-SIT1. The Omicron BA.5 variant of the SARS-CoV-2 Spike protein binds to the ACE2-SIT1 complex in HEK-293T cells, reducing the Pro-stimulated current by about 20%. The absence of Cl^-^ showed no effect, indicating that the Spike protein affects the transport activity of the ACE2-SIT1 complex [126].

ACE2 and collectrin help regulate NO and oxidative stress, supporting blood pressure balance and responding to vascular injury [127]. NO production is generated by NOS. Constitutively expressed endothelial NOS (eNOS) and neuronal NOS (nNOS) can only function properly when they are dimers, which requires factors like Arg, the cofactor BH_4_, and S-nitrosylation. Without Arg and/or BH_4_, NOS cannot produce NO, but they can create the superoxide anion (O_2_^•−^), a known phenomenon called “uncoupling” [116]. The effect of collectrin on NO production is thought to be related to its support of extracellular Arg transport by both Na^+^-independent and Na^+^-dependent Arg transporters, including B^0^AT1 [116], whereas ACE2 regulates NO production via the ACE2/Ang (1–7)/Mas pathway. The binding of Ang (1–7) to MasR induces stimulation of the PI3K/AKT pathway and NO production via eNOS. The cleavage of sACE2 induced by ADAM17 leads to increased production of ROS, especially O_2_^•−^ due to the intensified function of nicotinamide adenine dinucleotide phosphate oxidase (NADPH oxidase), as well as a notable increase in the degradation of nitric oxide [96].

### 4.9. Role of the ACE2 Endodomain

The ACE2 endodomain has predicted short linear motifs (SLiMs) that may facilitate interactions with proteins related to endocytosis, autophagy, and signaling (Figure 3B). Specifically, SLiMs were predicted to bind to class I PDZ domains (at AAs 796–805) and phosphotyrosine-binding (PTB) domains (AAs 787–798), and ACE2 SLiMs to Ser783 and Tyr781, which overlap with adaptor protein complex 2 (AP2) μ2, Src homology 2 (SH2), and the autophagy-related protein 8 (ATG8) domain of light chain isoform 3 of microtubule-associated protein 1 (MAP1LC3) and γ-aminobutyric acid receptor-associated protein (GABARAP). It has been suggested that ACE2 interacts with sorting nexin 27 (SNX27), Na^+^/H^+^ exchanger regulatory factor 3 (NHERF3), or SH3 and multiple ankyrin repeat domains 1 (SHANK1), but no functional evidence has been provided for this [128]. The NHERF protein family has four isoforms: NHERF1, NHERF2, NHERF3, and NHERF4. NHERF1 and NHERF2 have two PDZ domains each, while NHERF3 and NHERF4 have four PDZ domains. PDZ is an abbreviation for the first identified proteins with a similar structural domain: Postsynaptic density 95/Disc large/Zonula occludens-1 [129]. The distinctive recognition motif, 802QTSF805, which is present in human ACE2, is essential for enabling ACE2’s interaction with PDZ domain-containing proteins [129]. These proteins play a crucial role in the formation of macromolecular protein complexes at the plasma membrane [130]. An analysis of a PDZ domain library showed that the ACE2 PDZ binding motif binds, among other things, to neuronal proteins, SHANK, and Microtubule Associated Serine/Threonine Kinase 1 (MAST1)/MAST2 [131]. NHERF1 interacts with the ACE2 endodomain through its PDZ domains in several cell cultures, such as Calu-3 and Caco-2. Similar interactions have been reported for NHERF2. ACE2 interacts with both β-arrestin 1 and β-arrestin 2, regardless of NHERF1’s presence [129]. β-arrestins are endocytic adaptors and mediate the trafficking of various receptors to the cell surface [132]. They are thought to aid in the internalization of ACE2 via clathrin-mediated endocytosis, with NHERF1 acting as a scaffold protein that helps localize the ACE2-B^0^AT1 complex to the plasma membrane and control its internalization [129]. Bioinformatics predicted that ACE2 interacts with integrin β3, which can bind ATG8 in a phospho-dependent manner. This finding was validated through in vitro studies [128,133]. Integrin β1 has been found to increase ACE2 protein and mRNA levels in human and mouse kidney epithelial cells [134]. Integrins are adhesion molecules that can interact with a number of cytosolic proteins, mediating their interaction with the cytoskeleton. They can be activated both from the inside-out and the outside-in [133]. Research on HEK cells with high ACE2 levels shows that ACE2 interacts with ITGB1, enhancing its membrane attachment. In contrast, in Huh7 cells, the extracellular interaction between these proteins facilitated cell–cell adhesion [45]. Integrin α5β1, expressed on cell surfaces, played a pivotal role in facilitating ACE2-mediated entry of the Omicron strain of SARS-CoV-2 into susceptible cells [135], yet direct interaction with ACE2 remains unproven. Of note, the integrin–FAK signaling pathway is partly activated by integrins αν and β1, leading to Aβ-induced neurotoxicity in hippocampal neurons [136]. Tyr781 in ACE2 serves as a reliable phosphorylation site that interacts with multiple domains, depending on its phosphorylation. Phosphorylation prevents ACE2 from binding to AP2 μ2, while allowing it to attach to protein tyrosine kinase FYN’s SH2 domain [133].

### 4.10. Exosomal Full-Length ACE2

Exosomes from endothelial progenitor cells (EPCs) with elevated ACE2 levels safeguard endothelial cells that have been harmed by Ang II in vitro. They do this by increasing ACE2 levels, improving the Ang II/Ang (1–7) ratio, and enhancing mitochondrial function [137]. VSMCs can take up EPC-derived ACE2-loaded exosomes, primarily via caveolin-dependent endocytosis. This action reduced Ang II-induced VSMC phenotype changes, as shown by higher calponin and alpha smooth muscle actin (α-SMA) levels, lower cellular retinol-binding protein 1 (CRBP-1) and myosin heavy chain 10 (MYH10) levels, and decreased NF-κB. Thus, exosomal trafficking of functional ACE2 contributes to the suppression of the activated NF-κB pathway [138]. In aged mice, brain exosomes and their ACE2 and miR-17-5p showed a significant decrease compared with young mice. EPC-derived exosomes containing ACE2 and miR-17-5p reduced aging effects in older mice and senescent endothelial cells by decreasing ROS and apoptosis, while improving cell viability and tube formation. Silencing of miR-17-5p partially abolished the beneficial effects of exosomal ACE2. The protective effect of exosomal ACE2 was mediated by activation of the miR-17-5p/PTEN/PI3K/Akt signaling pathway [139].

### 4.11. The ACE2 Isoform 4

In addition to isoform 1, ACE2 has now been shown to express isoform 4, which is induced by IFNs [40,41,140]. In various studies, it has been designated as *deltaACE2* transcript (*dACE2*) [41], a truncated ACE2 isoform [40], or MIRb-ACE2 [140]. Notably, a study of the genome sequences of 100 vertebrate species only found this isoform in primates. It is obtained by splicing initiated by a new first exon in intron 9 of the *ACE2* gene [41] (Figure 2), which is located in the putative regulatory region of the *ACE2* promoter and contains several Interferon Response Sequences (ISREs) [40,41,140]. ISREs are responsible for the binding of transcription factors related to IFN signaling. This chromatin region bears H3K4me1, H3K4me3, and H3K27ac marks and has a DNase I hypersensitivity cluster [41]. In colonic and ileal organoid cultures, *dACE2* is expressed when treated with IFN-β or an IFNλ1–3 combination, but at lower levels than the full-length *ACE2* gene. However, the full-length *ACE2* consistently maintains its presence at baseline levels [41]. Type I IFN α/β/ω or type III λ initiates expression of IFN-regulated proteins through regulation of the ISRE element, whereas type II IFN γ works through regulation of the GAS element [141]. The *dACE2* promoter region is predicted to contain ISREs for all types of IFNs [40,41].

A study on the effects of different IFNs from SARS-CoV-2 infection in *Calu-3* and *Vero-CCL81* cells showed that IFN-λ1 significantly raised antiviral ISG levels. However, the increase in *ACE2* mRNA was minimal, and ACE2 protein levels were unchanged on cell surfaces. IFN-β greatly increased antiviral ISG and ACE2 levels at both the mRNA and protein levels, whereas IFNγ had the smallest impact on ISGs but raised ACE2 expression on the cell surface [142]. SARS-CoV-2 infection significantly induced *dACE2* and Interferon-Induced Protein with Tetratricopeptide Repeats 1 (*IFIT1*) gene expressions, but not *ACE2*. In the Cancer Genome Atlas (TCGA), *IFNG* is one of the most frequently expressed genes. While IFN-γ is not associated with *dACE2* in LUSC, it may elevate *dACE2* levels in specific tumors or infected sites due to increased levels of immune cells that generate IFN-γ [41].

In normal tissues, *dACE2* expression is usually absent or very low, likely due to the inflammatory tissue microenvironment [142]. An examination of normal tissues adjacent to the tumor found expression of both *ACE2* and *dACE2* in many tissue samples. *dACE2* had the highest expression in squamous cell carcinomas of the lung (LUSC), head and neck (HNSC), esophagus (ESCA), bladder (BLCA), and cervix (CESC) [41].

The truncated ACE2 protein is very unstable and seldom found in many cell lines, unlike its mRNA [40,41]. In primary brain culture astrocytes, more than 90% of ACE2 was full-length ACE2, with the remainder being expressed as dACE2 [35]. In addition, the ratio of expression levels of isoforms 1 and 4 in different organs can vary significantly [143].

In nasopharyngeal swab samples from SARS-CoV-2-infected individuals, *dACE2* mRNA levels were more elevated than *ACE2* mRNA levels [144].

IL-13 signaling alters *ACE2* gene expression and isoform ratios in primary human bronchial epithelial cells. In asthmatic patients and healthy controls, ACE2 isoform 1, glycosylated isoform 1, and isoform 4 were present in ratios of 1~1~1; isoform 3 was not expressed, and isoform 2 could not be identified or analyzed. IL-13 treatment reduced the expression of apical isoform 1 mRNA and N-glycosylation of full-length ACE2 protein. These downregulated genes were related to glycosylation, resulting in non-glycosylated full-length ACE2 remaining in the endoplasmic reticulum. IL-13 also decreased neuropilin 1 (*NRP1*) gene expression but increased dipeptidyl peptidase-4 (*DPP4*) and *TMPRSS2* gene expression. The main pathways that were upregulated in response to IL-13 were ion and transmembrane transport and lipid metabolism processes. The expression of *ACE2* isoform 4 mRNA was very stable, in contrast to protein stability. The expression of *ACE2* isoform 1 mRNA was found to show a trend of positive correlation with age, in contrast to *ACE2* isoform 4 mRNA [39]. It was reported that IL-13, along with IL-1α, IFN-γ, and GM-CSF, was increased in the mid-temporal cortex of AD patients [145]. Moreover, expression of IL-13 and IL-4 by reactive microglia may lead to neuronal death by regulating oxidative stress during dementia and AD [146].

## 5. The Impact of ACE2 on Selected Neurotransmitter Systems in the Brain

The examination of ACE2’s interaction with neurotransmitter systems is particularly intriguing, as these systems play a crucial role in brain function and are directly implicated in the development of Alzheimer’s disease and COVID-19. Studies indicate that ACE2 influences neurotransmitter dynamics by affecting their synthesis, degradation, release, and cellular uptake. This dynamic interaction affects the efficiency of synaptic transmission significantly. It is important to note that the activity of ACE2 itself depends on the state of neurotransmitter systems.

Currently, the most studied interactions of ACE2 are with the excitatory glutamatergic system and the closely related inhibitory GABAergic system. The balance between these neurotransmitter systems ensures normal activity of the neural network. Excessive Glu stimulation causes excitotoxicity, resulting in inflammation and neurodegeneration due to disrupted calcium balance and the initiation of various harmful signaling pathways, like mitochondrial dysfunction and oxidative stress. ACE2 is expressed in both glial cells and glutamatergic and GABAergic neurons. The decrease in GABAergic interneurons causes higher excitability, which might impair working memory and executive functions [17].

Glu induces TNFα production in the hippocampus of neonatal rats [147]. The high TNFα levels in the cerebrospinal fluid of hypertensive patients suggest that ADAM17 is active in the brain, leading to ACE2 shedding [82]. The hypothalamus has glutamate receptors [148]. Glutamate overstimulation in cortical and hypothalamic neurons raised ADAM17 activity, reducing mACE2 function through ACE2 shedding [147,148]. The use of ionotropic glutamate receptor antagonists or blockers of oxidative stress signaling pathways may mitigate the reduction in ACE2 enzymatic activity. This indicates a potential relation between glutamate-induced excitotoxicity and an imbalanced RAS [147].

Experimental insights reveal a complex link between the glutamatergic system and the RAS, as shown by reduced excitatory synaptic frequency in the basolateral amygdala after diminazene aceturate-triggered ACE2 activation. This decline could correlate with reduced expression of the NR1 subunits in the N-methyl-d-aspartate receptor (NMDAR-NR1) [149].

ACE2 regulates Glu and GABA transporters, playing a crucial role in the uptake and release of neurotransmitters in the synaptic cleft. Furthermore, ACE2 helps transporters reach the cell surface and aids in packing neurotransmitters into synaptic vesicles. Gln, Glu, and GABA can be transported into the cell by different subtypes of membrane transporters from the solute carrier (SLC) families, SLC1, SLC6, SLC7, and SLC38, which can be expressed in both neurons and astrocytes of the brain [150]. The transport of Gln into cells depends on the transporter type, with different types using antiport, uniport, or symport mechanisms. Gln can be exchanged for other amino acids, helping to regulate the levels of Gln and amino acids inside cells [150]. As noted previously, ACE2 can form tetraheteromeric proteins with the amino acid transporters B^0^AT1, B^0^AT3, and SIT1 [108]. Importantly, B^0^AT1 can be a transporter for Gln [115], and SIT1 is thought to transport Pro for Glu synthesis in brain cells [108]. Physiologically, Pro can act as a weak agonist of glycine and ionotropic Glu receptors. Neurologically, SIT1 regulates Pro concentrations by modulating glycine and NMDA-type glutamate receptor activity in mice [113]. B^0^AT3 is present in synaptic vesicles, transporting Gln in GABAergic and other neuronal types [151,152]. In neurons, Gln is converted to Glu, which is refilled into synaptic vesicles. Glu transporters consist of five plasmalemmal excitatory amino acid transporters, EAAT1 (encoded by the gene *SLC1A3*), EAAT2 (*SLC1A2*), EAAT3 (*SLC1A1*), EAAT4 (*SLC1A6*), and EAAT5 (*SLC1A7*), along with two neutral amino acid transporters, ASCT1 (*SLC1A4*) and ASCT2 (*SLC1A5*). Glu transport is primarily mediated by EAAT transporters, but at a low pH, it is mediated by ASCT [153]. Astrocytes take up about 90% of the Glu that is released in the CNS using the glial transporters EAAT1 and EAAT2. They are also capable of synthesizing any remaining Glu de novo or reclaiming it by recycling Gln from γ-aminobutyric acid (GABA) during the reuptake process [154]. Astrocytes convert Glu from the synaptic cleft into Gln, which is then sent back to neurons through SLC38 transporters. These transporters function as glutamate–glutamine shuttles, effectively preventing extracellular Glu-induced excitotoxicity [153]. SLC6A7 encodes the Pro transporter (ProT), an AA that plays a critical role in several metabolic pathways, including mitochondrial Arg and Glu synthesis. Extracellular GABA concentrations are kept at low levels thanks to the high-affinity Na^+^/Cl^-^-dependent GABA transporters GAT1-GAT3, which are encoded by the *SLC6A1*, *SLC6A13*, and *SLC6A11* genes. GAT1 is primarily found in the axonal terminals of GABAergic neurons, while GAT3 is found in glial cells. The vesicular GABA transporter, known as VGAT or vesicular inhibitory amino acid transporter (VIAAT), is responsible for replenishing synaptic vesicles with Gly, GABA, or β-Ala in both the brain and spinal cord [153]. Vesicular transporters (VGLUT 1–3) package Glu, but not Asp or Gln, into synaptic vesicles and transport it to the membrane. In the brain, VGLUT expression is predominantly localized to astrocytes in the hippocampus, cerebral cortex, and cerebellum, where it is found in vesicular structures that are distributed throughout the cytoplasm [153].

Using two different ACE2 blockers, it was found that it was possible to control the trafficking of Glu and GABA transporters, and that a decrease in their activity can seriously affect the uptake of the mediator from the synaptic cleft. Research indicates that the reservoir of Glu and GABA transporters is situated within exocytosis-like vesicles, which are responsible for transporting these proteins to the plasma membrane at the presynaptic level. This mechanism is crucial for the uptake of Glu and the exocytotic release of GABA. The functional modulation of the cytoplasmic tail of ACE2 can initiate numerous cellular signaling pathways, including the activation of PKC, which regulates the cell surface expression of GABA transporters. The impairment of PKC-mediated regulation of transporter expression on the cell surface led to a significant reduction in the uptake of Glu and GABA, as well as a decrease in GABA exocytosis. All the above-described disturbances indicate that ACE2 has effects on GABA-Glu systems that are not associated with the RAS. The inhibition of Glu and GABA transporters disrupts their normal flow, resulting in their buildup in the synaptic cleft. This accumulation instigates excitotoxicity and disturbs the balance between the brain’s excitatory and inhibitory systems, which is a key feature of certain types of AD and particularly evident in COVID-19 [155].

The *GAD1* and *GAD2* genes encode the glutamic acid decarboxylase enzymes GAD67 and GAD65, respectively, which are involved in GABA synthesis from Glu [156]. The ACE2/Ang (1–7)/Mas pathway plays a crucial role in governing glycolipid metabolism across various tissues, enhancing GABAergic signaling expression in response to metabolic stress [157]. In animal studies examining liver insulin resistance, Ang (1–7) has been demonstrated to enhance both the mRNA and protein levels of glutamate decarboxylase GAD67 [158]. In the cerebral cortex, GABA is produced by GAD67 and GAD65. The levels, locations within cells, and functions of these enzymes differ among various subpopulations of interneurons. GAD67 is present in various subcellular compartments, while GAD65 is mainly localized at synaptic terminals, connecting the synthesis of GABA to its transport into synaptic vesicles. GAD67 plays a crucial role in producing the majority of GABA in the brain under typical conditions, whereas GAD65 becomes vital during periods of increased synaptic activity [159]. A study on APP/PS1 transgenic mice exhibiting an epileptic phenotype revealed that the loss of parvalbumin interneurons, which are crucial inhibitory cells in the hippocampus, is linked to disruptions in neuregulin 1 (NRG1)-ErbB4 signaling. Diminished inhibitory synaptic transmission, stemming from reduced levels of GAD65/67 and GABA transporters, significantly contributes to the imbalance between excitation and inhibition, a key factor in the AD pathogenesis [160]. In cortical interneurons, BDNF/TrkB signaling controls the activation of GAD65 transcription in a RAS-ERK-CREB-dependent manner [159]. BDNF plays an important role in modulating synaptic plasticity, memory, and cognitive function. In the CNS, BDNF is primarily released from the hippocampus and hypothalamus, while peripheral BDNF is secreted by various tissues and platelets and is unable to cross the BBB [26]. ACE2 is one of the main proteins regulating BDNF release. The ACE2/Ang (1–7)/MAS axis regulates several downstream signaling cascades, in particular PI3K/Akt1/CREB/BDNF/TrkB [17].

ACE2 is vital for maintaining balance between the GABA and glutamate systems, which can be disrupted in conditions like Alzheimer’s disease. This disruption is characterized by the inability of astrocytes to effectively reuptake glutamate, compounded by mutations in genes encoding NMDA and AMPA glutamate receptor subunits, which results in significant changes in ion channel activity [160]. Based on this, maintaining ACE2 activity could be the basis for therapeutic interventions in AD.

The activity of the RAS and ACE2, in particular, is linked in many ways to the dopaminergic system in the brain. The dysregulation of the RAS leads to the deterioration of dopaminergic neurons in the nigrostriatal system, triggering the activation of AT1R [161]. This activation initiates the cellular NADPH oxidase–superoxide pathway and enhances Ca^2+^ release, which is essential for numerous processes linked to oxidative stress and neuroinflammation. Crucially, AT1R forms a complex with ACE2 that breaks apart as Ang II levels increase. This complex is eventually internalized from the cell surface into the endosome, which leads to the degradation of ACE2 within lysosomes [44]. Dopamine, via D2 receptors, suppresses angiotensinogen production and increases AT2R expression in astrocytes. In microglia, dopamine administration causes a decrease in the AT1R/AT2R ratio and inhibits the inflammatory response [162]. Research has revealed the presence of Ang II receptors alongside NADPH oxidase components in dopaminergic neurons and glial cells, particularly microglia, within the substantia nigra (SN) across various mammalian species, including humans [163,164,165]. This suggests that Ang II could exert a direct influence on these receptors, thereby modulating the neuroinflammatory response. In astrocytes, a reduction in dopamine levels leads to increased paracrine release of angiotensinogen/Ang II, which can have an effect on both neurons and microglial cells [163,164,165]. Furthermore, AT1R mediates the activation of the microglial inflammasome complex in dopamine depletion models [166]. Research has revealed that AT1R pairs with dopamine D2 receptors, and, intriguingly, both AT1R agonists and antagonists have the ability to selectively modify the functional responses of D2 receptors [167]. Ang II has been demonstrated to influence the expression and axonal trafficking of critical enzymes that are involved in catecholamine biosynthesis, particularly tyrosine hydroxylase and dopamine β-hydroxylase, ultimately regulating the production of norepinephrine and dopamine [168]. Studies show that there is a notable age-related decline in the expression of essential components of the anti-inflammatory RAS axis, particularly AT2R and MasR. This decline occurs not only at the cell membrane level but also within the intracellular RAS [169]. The intracellular RAS can compensate for the detrimental effects of plasma membrane AT1R activation. The internalization of the Ang II/AT1R complex activates nuclear AT1R, initiating protective mechanisms that combat oxidative stress caused by cell membrane AT1R [169].

It should be noted that data on the direct interaction of ACE2 with dopamine is very limited. Studies reveal that the dopa decarboxylase (*DDC*) gene [170], which plays a vital role in the synthesis of dopamine and serotonin, shows a remarkably strong statistical correlation in co-expression with *ACE2*. SARS-CoV2-induced downregulation of ACE2 expression can reduce the synthesis of dopamine and serotonin (5-HT), causing hypodopaminergia [171]. At the same time, the SARS-CoV-2 viral load was positively associated with *ACE2* expression and negatively correlated with *DDC* and *dACE2* [144]. Notably, patients with Parkinson’s disease (PD) and PD models exhibit elevated levels of autoantibodies targeting ACE2 and AT1R. These antibodies play a vital role in undermining the integrity of the BBB, are associated with the degeneration of dopaminergic neurons, initiate neuroinflammatory responses, and ultimately increase AT1R expression, while simultaneously inhibiting the ACE2-mediated anti-inflammatory RAS pathway. Plasma obtained from individuals who have recovered from COVID-19 has been shown to contain ACE2 autoantibodies, resulting in reduced endogenous sACE2 activity and hindering the effectiveness of exogenous ACE2 [172]. Several studies have shown counter-regulation between angiotensin and dopamine receptors in the SN and striatum [173,174]. Thus, dysregulation of RAS/dopamine interactions in the nigrostriatal system contributes to neuroinflammation and dopaminergic neurodegeneration [162,173].

Decreased ACE2 levels may significantly affect the functioning of the serotonergic system, which is involved in emotional responses, memory, and neurogenesis. The ACE2-B^0^AT1 complex plays a critical role in transporting Trp, a precursor for serotonin (5-HT), which explains the dramatic 70% reduction in plasma Trp levels that has been observed in *ace2*-null mice [175]. 5-HT levels were also reduced in the brain and blood of ACE2-deficient mice [176]. Research indicates that mutations in the *SLC6A19* gene can disrupt the B^0^AT1 transporter, resulting in the onset of Hartnup disease in humans [119]. Decreased ACE2 levels in patients with SARS-CoV-2 caused Trp depletion, leading to 5-HT deficiency [177].

The RAS, and specifically ACE2, plays a crucial role in the acetylcholinergic system, which is integral to numerous behavioral responses such as learning and memory, attention and arousal, as well as involuntary muscle contractions. In the brain, acetylcholine (ACh) functions as a neurotransmitter and a neuromodulator. It acts on two main types of receptors: nicotinic nAChRs and muscarinic mAChRs. Notably, in the lung, kidney, circulatory system, and brain, nAChRs are often localized on the same cells that express ACE2. It has been shown that nicotine can activate ACE2 in neurons, glia, and endothelial cells through nAChR activation. Importantly, stimulation of nAChRs can increase the release of several neurotransmitters, such as Glu, GABA, and dopamine. Therefore, nAChR plays a central role in synchronizing neuronal activity [178].

A possible interaction of ACE2 with α7nAChR was detected in U373 cell lysates, and α7AChR activation was shown to effectively inhibit SARS-CoV-2 interaction with ACE2 [179]. Interestingly, ApoE4 significantly reduces ACE2 protein expression in vitro and in vivo, subsequently reducing the conversion of Ang II to Ang (1–7), and also induces degeneration of the cholinergic system [180,181]. Novel molecular mechanisms linking cholesterol metabolism to ApoE ε4 have been identified. In an in vitro study of cholinergic neurons, the expression of ApoE ε4 led to an increase in intracellular cholesterol levels while simultaneously downregulating the associated gene, which resulted in a decreased availability of acetyl-CoA, which is necessary for acetylcholine synthesis. This information paves the way for a deeper exploration of how ACE2 impacts the acetylcholine system by dysregulating ApoE ε4-dependent cholesterol metabolism, ultimately resulting in neurotoxicity and neuronal death [181].

Glu excitotoxicity emerges at the intersection of the RAS, neurotransmitter systems, and kallikrein–kinin hormonal system, highlighting their critical roles in the progression of Alzheimer’s disease [182]. Elevated levels of Glu can result in heightened production of the ADAM17 protein and increased shedding, mediated by ADAM17. This also results in a reduction in ACE2 activity in primary hypothalamic neurons, accompanied by an increase in kinin receptor B1 (B1R) expression, while kinin receptor B2 (B2R) remains unaffected [148]. The vasoactive kinin peptides within the kallikrein–kinin system play a crucial role in modulating inflammation and regulating blood pressure by activating their two main receptors, B1R and B2R. The endogenous B1R ligands DEABK and Lys-(Des-Arg9)-bradykinin (LDEABK) are metabolites of bradykinin and kallidin, respectively. B1R expression is tissue-specific and strongly induced by inflammation or oxidative stress, activating B1R-mediated signaling pathways. ACE2 inactivates LDEABK and DEABK, rendering them unable to bind to B1R. Both kinin receptors are expressed in neurons; in particular, B1R expression has been detected in neurons of the human thalamus, hypothalamus, and spinal cord, and in dopaminergic neurons of the ventral tegmentum of spontaneously hypertensive rats. Unlike B2R, B1R remains on the cell surface following agonist stimulation, as it instead translocates and aggregates in response to agonist binding, ultimately amplifying B1R-mediated responses [148,161]. Ang II treatment of neurons resulted in enhanced B1R expression through the activation of AT1R. B1R, in turn, decreased mitochondrial respiration and led to an increase in the expression of two subunits of NADPH oxidase (Nox2 and Nox4), IL-1β, IL-6, and TNFα genes, increased oxidative potential, and DNA binding activity of NF-kB p65. The roles of AT1R and B1R in neuroinflammation and neurogenic hypertension are well established, yet the nature of their direct interaction in Ang-II-induced neuroinflammation and the resultant oxidative stress in neurons remains uncertain [161]. BRB1 receptor (B1R) overactivation promotes inflammation and coagulation [12].

## 6. ACE2 as a Cell Receptor for SARS-CoV-2 Entry

Unlike other pathogenic human coronaviruses (HCoV), which cause mild respiratory diseases in humans, SARS-CoV, MERS-CoV, and SARS-CoV-2 viruses from the genus *Betacoronavirus* can cause severe respiratory syndrome [8,9]. However, they use different receptors to enter the cell: SARS-CoV and SARS-CoV-2 use ACE2, and MERS-CoV uses dipeptidyl peptidase-4 (DPP4). During the COVID-19 pandemic, SARS-CoV-2 demonstrated the ability to infect cells lacking ACE2 by utilizing alternative receptors, including CD147, AXL, CD209L/L-SIGN/CLEC4M, CD209/DCSIGN/CLEC4L, CLEC4G/LSECtin, KREMEN1, ASGR1/CLEC4H1, LDLRAD3, TMEM30A/CD50A, Clec4g (mouse), and CD209c (mouse). Nevertheless, ACE2 remained the key receptor during the entire course of the infection [9]. ACE2 is utilized as a receptor by HCoV-NL63 from the genus *Alphacoronavirus*, yet it does not trigger severe acute respiratory syndrome [12]. This is likely attributable to variations in the molecular mechanisms that are responsible for disease progression. Notably, the adhesion of HCoV-NL63 does not trigger the activation of ADAM-17 or the release of sACE2 [22].

The main mode of transmission of SARS-CoV-2 is airborne droplets [10]. Numerous studies indicate that the virus might infiltrate the brain through various pathways: (1) via the sensory neurons of the olfactory bulb, potentially accounting for the clinical symptoms of anosmia and hypogeusia that were observed in some COVID-19 patients; (2) through the vascular system; and (3) via the lymphatic system when the BBB is compromised. It originally spread through systems that were directly or indirectly connected to the olfactory network, which encompasses the piriform cortex, basal ganglia, midbrain, and hypothalamus. Subsequently, it extended its influence to the substantia nigra within the midbrain, as well as the amygdala, hippocampus, and cerebellum. Notably, there was initially massive neuronal death, but no evidence of severe neuroinflammation, astrogliosis, or microglial activation, and only at a late stage did pronounced hyperproduction of proinflammatory cytokines likely contribute to the high mortality [18,27,29]. Upon the virus invading the nasal epithelium and olfactory bulb, it subsequently impacted the frontal lobe, primarily due to the elevated presence of ACE2 [18,29]. The intercellular transmission of the virus was mediated by exosomes released from infected cells, causing it to spread from neuron to neuron and ultimately leading to the demise of neuronal cells. The death of microglial cells is likely a consequence of the activation of the mitochondria-dependent intrinsic apoptotic pathway, triggered by the Spike protein of SARS-CoV-2 [18]. Spike proteins (S1 and trimer) also caused mitochondrial damage in brain endothelial cells [32]. Furthermore, the disruption in the equilibrium of Ang II and Ang (1–7) levels caused by SARS-CoV-2 infection resulted in a reduction in pyruvate dehydrogenase complex activity, ultimately impairing cellular energy metabolism [11]. Thus, the death of several types of brain cells contributed to the development of neurological symptoms in some COVID-19 patients, and the difference in the damage to various brain regions and the locations of persistent SARS-CoV-2 virus in the brain after the acute phase of infection [18] are associated with different routes of virus penetration into the brain, as well as pre-existing brain diseases. At the same time, many noted signs of accelerated brain aging, similar to those observed in AD [17,18,183].

SARS-CoV-2 virion particles, much like those of SARS-CoV, bind to ACE2 via a structural glycoprotein known as the Spike or S protein, trimers of which are located on the spherical surface of particles. The majority of amino acid residues in the receptor-binding domain (RBD) of the Spike proteins from both viruses remain conserved. However, certain amino acid changes were observed in SARS-CoV-2 protein variants that utilize alternative receptors [8,50]. The S proteins of these two viruses share roughly 76% amino acid similarity, yet five amino acid alterations in the RBD of SARS-CoV-2 improve its attachment to ACE2 significantly compared with SARS-CoV [8,184]. Each ACE2 monomer within the dimer has the capability to bind to the S protein trimer [93]. The critical ACE2 residues that are involved in the interaction with the SARS-CoV-2 RBD are Gln 24, Asp 30, His 34, Tyr 41, Gln 42, Met 82, Lys 353, and Arg 357 [38].

Glycan–glycan interactions are often the first step in viral adhesion to cells when they possess the appropriate carbohydrate structures [185]. In a manner akin to ACE2, the S protein functions as a glycoprotein, exhibiting a wealth of N- and O-glycosylation sites; furthermore, its glycans have been found to interact with the glycans in hACE2 [93,112,186].

Priming of S protein trimers by host cellular proteases is required for viral entry into cells. In addition to TMPRSS2, SARS-CoV2 S protein can prime a wide range of host proteases, such as furin, cathepsin L, cathepsin B, trypsin, factor X, and elastase, due to the presence of additional cleavage sites [8,22,187,188,189] and whether the cell expresses TMPRSS2 [190]. Upon priming, SARS-CoV-2 ProS monomers form two subunits, S1 and S2, which are held together by non-covalent interactions. The S1 subunit, which carries an RBD at the N-terminus, then changes its conformation, which previously allowed the virus to escape recognition by the immune system. This alteration enables the exposed RBD to effectively bind with ACE2 receptors that are present on the cell surface [187,190]. The S2 subunit is responsible for the fusogenic activity of the S protein [190]. Upon binding to ACE2, SARS-CoV2 virions can transfer their viral genetic material into host cells. The fusion of the viral envelope with the host’s cytoplasmic membrane can take place after TMPRSS2 priming, or priming may occur later inside the endosome if the target cell is deficient in TMPRSS2 or if the virus–ACE2 complex fails to properly interact with TMPRSS2. In these scenarios, the virus that is bound to ACE2 is absorbed into the late endolysosome via clathrin-mediated endocytosis, where it is activated by a range of other proteases [13,50,190]. Initially, it was suggested that the ACE2 protein undergoes internalization during SARS-CoV-2 infection through clathrin-mediated endocytosis and is subsequently recycled back to the cell surface [44]. Research utilizing a pseudovirus along with isolated SARS-CoV-2 Spike protein has revealed that the downregulation of ACE2 does not stem from proteasomal degradation linked to the ubiquitination of ACE2 at Lys788 by MDM2. Rather, the process is prompted by clathrin-mediated endocytosis following the attachment of the S protein, leading to degradation inside the lysosome in both hamster lung tissue and human HEK293A cells. The AP2 binds to the potential endocytic sorting motif 781YASI784, which is found in the cytoplasmic section of ACE2, instigating its clathrin-mediated endocytosis together with the S protein. The downregulation of ACE2 was primarily caused by the binding of the S protein, rather than by the replication of the virus. HEK293A cells treated with the SARS-CoV-2 S protein and those with ACE2 knockdown exhibited comparable changes in downstream gene expression, impacting genes that are involved in interleukin and cytokine signaling pathways, as well as the JAK-STAT and MAPK signaling pathways, along with the AP-1 transcription factor network. This indicates that the downregulation of ACE2 activates these signaling cascades [191]. HEK293T, Calu3, and Caco-2 cell lines also demonstrated that the ACE2 Spike protein was co-endocytosed and degraded as a complex in the cells. The engagement of ACE2 with the viral RBD alone was enough to trigger its degradation. This process occurred through the autophagosome–lysosome pathway, where the full-length Spike protein was specifically cleaved within lysosomes. The level of ACE2 mRNA in cells did not change [192].

Endocytosis demands cytoskeletal remodeling, in particular driven by P21-activated kinase 1 (PAK1), a pivotal activator whose phosphorylation is performed by protein kinase CK2α [192]. CK2 was one of several host cell kinases, alongside CMGC, CDK, PKC, PIKFYVE, EIF2AK2, ATM, and CHEK1, that exhibited upregulation due to SARS-CoV-2 infection. SARS-CoV-2 infection leads to changes in the phosphorylation of various host proteins, significantly impacting multiple signaling pathways, such as MAPK, EGFR signaling, TGF-β, autophagy, and AKT [193]. Additionally, the activation of several known tau-targeted kinases, including AMPK, glycogen synthase kinase 3 beta (GSK3β), protein kinase A (PKA), and calcium/calmodulin-dependent protein kinase II (CAMKII), was detected in the brains of COVID-19 patients [17].

According to various studies, the susceptibility of cells to SARS-CoV-2 infection differs based on the type of cell culture that is utilized. Research reveals that the virus primarily targets neurons, whereas astrocytes originating from human induced pluripotent stem cells (iPSCs) are largely resistant to infection [189]. SARS-CoV-2 entered human iPSC-derived neurons via the endosomal route, and its entry was independent of TMPRSS2 [189]. Conversely, in isolated from CD-1 mice primary astrocytes, neurons, and brain microvascular endothelial cells, the virus primarily targets astrocytes [35].

It is also noteworthy that the RBD of SARS-CoV-2 S1 protein increases ACE2 peptidase activity significantly, which is not observed in SARS-CoV, MERS-CoV, or HKU1 [186].

## 7. Is Alzheimer’s Disease a Comorbidity or a Consequence of COVID-19-Induced Neuropathology?

Neurodegenerative diseases, such as AD, typically arise from a complex interplay of environmental and genetic factors. It is crucial to acknowledge that infectious agents, such as viral and bacterial toxins that affect the CNS, contribute significantly to the neurodegeneration process [18]. The intricate mechanisms through which COVID-19 impacts the brain involve both direct viral effects and indirect systemic responses to the disease [18]. Nearly 35% of all COVID-19 patients experienced neurological and neuropsychiatric symptoms and manifestations [31], such as headache, decreased levels of consciousness, altered mental status, encephalopathy-like features, Guillain–Barré syndrome, meningoencephalitis, neuralgia, hyposmia, hypogeusia, and stroke [9,31]. Alzheimer’s disease in these patients rendered them more prone to SARS-CoV-2 infection, while also significantly raising their risk of mortality [34,194,195]. This could be a result of amplified sACE2 in the brain and a loss of ACE2 protective function due to diminished mACE2 levels. Postmortem brain studies have revealed a significant increase in the expression levels of ACE2 in the brain tissue of individuals who were diagnosed with AD in comparison to healthy controls [7,196,197]. sACE2 was detected in the cerebrospinal fluid of COVID-19 patients with encephalitis, while those without encephalitis showed normal levels of ACE2 and TMPRSS2. The increasing level of sACE2 with catalytic activity probably reflected blood–brain barrier disruption and the development of encephalopathy [198]. Some studies have demonstrated a decrease in mACE2 expression and activity levels [27,199]. These discrepancies are likely related to both the AD subtype [30,200] and disease stage [201], as well as the presence of diseases such as arterial hypertension, myocardial hypertrophy, and chronic heart failure [27]. A study examined the expression of key RAS genes and the Leucyl and Cystinyl Aminopeptidase (*LNPEP*) gene in postmortem frontal cortex brain tissue from individuals with various forms of dementia—specifically, (a) AD, (b) vascular dementia, and (c) AD coupled with vascular dementia—alongside samples from individuals experiencing normal aging. The findings revealed that the expression levels varied significantly depending on the stage of the disease [30]. In the process of normal aging, heightened signaling from *ACE1/AGTR1* expression is predominantly countered by *ACE1/AGTR2* expression, while the levels of *ACE2*, *MAS1*, and *LNPEP* remain constant, while in the later stages of Alzheimer’s disease, a decline in *MAS1* expression emerges. This indicates a potential reduction in the regulatory function of the RAS. Studies show that the expression of the *MAS1* gene, primarily located in neurons and astrocytes, is inversely related to the concentrations of Aβ and tau. This expression also varies between patients who are diagnosed with AD and those with concurrent AD and vascular dementia. A rise in *LNPEP* expression was solely linked to the progression of vascular dementia [30]. In addition, as shown in vitro in mouse brain lysates, both key RAS enzymes, ACE and ACE2, can process neurotoxic proteins Aβ43 and Aβ42, which are involved in the pathogenesis of AD. ACE2 enables the conversion of Aβ43 to Aβ42, and when paired with ACE, it can change Aβ43 into Aβ40, consequently suppressing amyloid deposition [202]. Therefore, a decrease in ACE2 activity promotes Aβ deposition.

In Alzheimer’s disease, the accumulation of extracellular Aβ can lead to cerebrovascular damage and reduced cerebral blood flow, as well as disruption of the BBB, long before cognitive decline is observed. The BBB consists predominantly of endothelial cells, with tight junction proteins (TJPs) such as claudins, occludins, and zonula occludens (ZO) positioned between them, along with pericytes and astrocytes. ZO forms a connection with the PDZ motif in the intracellular domains of claudins and occludin, integrating with the actin cytoskeleton to support the structural integrity of tight junction proteins. Aβ25–35 inhibits ACE2 expression in endothelial cells, significantly reduces ZO-1 protein levels, and activates NF-κB pathways. NF-κB activation extensively promotes VEGF transcription, sparking angiogenesis. However, in the AD context, this phenomenon results in both new blood vessel formation and increased blood vessel permeability, further compromising the integrity of the BBB. In an in vitro AD model, the overexpression of ACE2 was demonstrated to mitigate pathological angiogenesis and diminish BBB damage by inhibiting the activity of the NF-κB/VEGF/VEGFR2 pathway and restoring the expression of ZO-1 and claudin-5 [199]. A notable rise in BBB permeability, paired with a substantial decline in the expression of ZO1, VE-cadherin, and occludin proteins, yet sparing claudin-5, has facilitated the entry of SARS-CoV-2 into the brain [9]. The Spike protein of SARS-CoV-2 has demonstrated its ability to compromise the BBB, leading to neuronal damage either directly or indirectly through the activation of brain mast cells and microglia. The activation of this process results in the release of multiple neuroinflammatory molecules, which subsequently instigate astrogliosis and the buildup of amyloid deposits [203].

ACE2 shedding has been observed in acute respiratory syndrome in both SARS-CoV and SARS-CoV-2 infections [204,205]. In a postmortem study of AD brains, the level of sACE2 was increased in the parietal cortex of AD patients. High sACE2 levels from the cerebrovascular fraction were negatively correlated with antemortem global cognitive performance. These trends were most pronounced in AD patients carrying the *ApoE4* allele. In contrast, mACE2 was negatively correlated with clinical, neuropathological, and vascular biomarkers of AD. Recent findings suggest that the release of ACE2 from membranes is correlated with pericyte dysfunction at the BBB, a phenomenon that appears to be more associated with the emergence of neurodegenerative disorders than with aging [7]. In addition, there is a crucial causal relationship between genetically elevated circulating sACE2 levels and an increased AD risk [206].

Age stands out as the most significant factor among the various physio-pathological elements leading to the progression of AD, along with the genetic variant *APOE4* and the activation of intracellular pathways that are linked to abnormal APP/Aβ and tau metabolism [17].

ApoE is a protein with a molecular weight of 34 kDa (299 AAs). The structure features a receptor-binding region (136–150 AAs) in the N-terminal domain and a lipid-binding region (244–272 AAs) in the C-terminal domain, united by a flexible hinge region (167–206 AAs) [207]. In humans, there are four identified single-nucleotide polymorphisms in the *APOE* gene, which give rise to three distinct allelic variants: ε2, ε3, and ε4. These variants correspond to three distinct protein isoforms: E2, E3, and E4 [208,209]. The substitution of Cys with Arg at position 112 in APOE3 and APOE4 leads to notable changes in these proteins’ structure and function, primarily by altering the interactions between their domains. As a consequence, APOE4 is more sensitive to proteolytic cleavage, accompanied by the formation of neurotoxic protein fragments [207]. The ε4/ε4 genotype is strongly associated with an increased likelihood of developing both sporadic and familial late-onset Alzheimer’s disease [208,209]. In contrast to APOE4, APOE2 has a protective effect against AD [209]. The *APOE* ε3 genotype is the most prevalent, with an impressive global frequency of approximately 78%. In comparison, *APOE* ε4 is found in about 10% to 20% of different populations, while *APOE* ε2 has an average occurrence of around 8% [209].

The APOE protein plays a crucial role in the metabolism and transport of lipids and cholesterol. In addition to its main production in hepatocytes, ApoE is synthesized by various cell types, such as astrocytes, glial cells, and neurons, particularly in reaction to aging and stress [208,210]. In the brain, astrocytic ApoE is lipidated and transported to other cell types. Furthermore, as shown in cell culture, the fate of internalized ApoE differs in different cell types. In neuroblastoma and astrocyte cells, ApoE predominantly accumulates in lysosomes, whereas in neurons, it is primarily found in neurite endosomes and autophagosomes, showing no presence in the neuronal soma. This indicates that in neurons, ApoE is not subjected to lysosomal transport and degradation [210]. Internalized ApoE and APP and/or Aβ can cross-link and interact within endosomes. ApoE4 is thought to influence Aβ42’s internalization, subsequent aggregation, and/or degradation in neurons [210]. Astrocytic ApoE is thought to be involved in the Aβ pathology, whereas neuronal ApoE has a more pronounced effect on neuronal function, survival, and the development of neurofibrillary tangles. ApoE is believed to facilitate the breakdown of Aβ by modifying its structure, making it more identifiable to proteolytic enzymes. The various ApoE isoforms play a crucial role in the effectiveness of Aβ clearance during the later stages, with ApoE2 leading the way as the most efficient, followed by ApoE3, and ApoE4 trailing behind. This variation may be partly attributed to the increased susceptibility of ApoE4 to proteolytic breakdown. GABAergic interneurons within the dentate gyrus are especially susceptible to neurotoxicity driven by the ApoE4 fragment [207]. A proposed model outlines how the development of Alzheimer’s disease in APOE ε4 carriers occurs. Studies reveal that the loss of GABAergic interneurons in the hippocampus results in network dysfunction and increased hyperexcitability, both of which significantly impair learning and memory. Furthermore, this dysfunction induces additional stress that heightens the neuronal expression of apoE4. As a result, further loss of GABAergic interneurons and, ultimately, cognitive decline occur [207]. Overall, this is consistent with the fate of GABAergic, particularly PV+, interneurons in specific brain regions that are preferentially affected in AD [211] and the Glutamate/GABA system imbalance caused by GABAergic transmission impairment that drives AD with age [154,160]. Notably, patients with COVID-19 also showed impaired GABAergic neurotransmission. It is believed that GABA and GABA agonists may effectively treat COVID-19 by reducing proinflammatory cytokines and blocking inflammatory pathways like NF-κB and the NLRP3 inflammasome [16]. Studies on COVID-19 have uncovered a correlation between the severity of the disease and an impaired reverse cholesterol transport mechanism in individuals with the *APOE* ε4/ε4 genotype [180,208]. High intracellular cholesterol boosts cholesterol-rich membrane lipid rafts, increasing ACE2/TMPRSS2 levels and potentially making cells more susceptible to SARS-CoV-2 infection [208]. Research shows that APOE4 is much less effective than APOE3 at blocking ACE2 from interacting with the Spike protein. This makes it harder for the SARS-CoV-2 pseudovirus to enter cells. This difference could potentially impact the severity of COVID-19 in individuals carrying the *APOE* ε4/ε4 genotype [209]. APOE has the ability to directly interact with ACE2. Although the interactions lacked a specific isoform binding pattern, studies with ApoE-TR mice showed that they were dose-dependent. Molecular docking and simulation analyses have identified promising binding sites for ApoE2, ApoE3, and ApoE4 on ACE2, specifically localized at N53-K68, L45-K68, and N330-R357, respectively. Notably, these sites coincided with the interaction region for the S1 component of the SARS-CoV-2 Spike protein. ApoE4-TR mice had increased Ang II and reduced Ang (1–7) levels in their cortex, kidney, and intestine compared with other ApoE types. The study revealed a substantial increase in the binding of ApoE2, ApoE3, and ApoE4, leading to a disruption in the brain’s RAS as a result of ACE2 deficiency [180]. A new discovery emphasizes that the middle and C-terminal segments of the ACE2 zinc domain play a significant role in the interaction between ACE2 and APOE3 in vitro [209]. Research shows that APOE4 interacts with ACE2 and reduces ACE2 protein levels both in vitro and in vivo. Researchers did not find any inhibitory effects in the lung tissues of ApoE-TR mice after examining several organs, including the cerebral cortex, hippocampus, liver, intestine, kidney, heart, and lungs. However, a significant inhibitory effect was noted in the cortex, especially within the hippocampus. These results were consistent with the ACE2 expression data in AD patients carrying the APOE ε4 allele. APOE ε4 carriers show several changes that might contribute to asthma development after a SARS-CoV-2 infection. These factors involve the promotion of BBB dysfunction, the heightened secretion of proinflammatory cytokines by peripheral macrophages and microglia in the CNS, and the downregulation of several proteins linked to ISGs [17].

Alzheimer’s disease is traditionally seen as marked by the accumulation of Aβ peptide plaques outside nerve cells and hyperphosphorylated tau tangles inside nerve cells, especially in the cerebral cortex and hippocampus. This accumulation leads to neuronal and synaptic damage, paving the way for cognitive decline. In-depth studies of AD have uncovered its diversity, which causes different symptoms and indicates the need for specific treatments for each subtype. The study of the transcriptome in patients with sporadic late-onset AD (LOAD) made it possible to identify three large classes of molecular signatures (A, B, and C), where class B included subtypes B1 and B2, and class C included subtypes C1 and C2. All AD subtypes were conserved and did not depend on age or disease severity [200]. Classes A + B were characterized by predominant activity of microtubule-associated protein tau (MAPT), while class C was characterized by predominant activity of the Aβ binding, clearance, and fiber formation pathways. Classes A and C showed opposite gene expression in AD-related signatures, which may lead to either inhibitory or excitatory dysfunction in some neuronal functions. Patients with class C Alzheimer’s disease exhibited significant immune system issues, such as reactive gliosis and BBB disruption, especially in the C1 subtype that is linked to *APOE4* alleles. Class C is characterized by increased expression of *TLN1*, *MSN*, and *IL6R* genes in microglia, and its subtypes are thought to be driven by inflammatory processes [200]. The reported abnormalities in the course of COVID-19 and long COVID show that they have strong similarities with those observed in AD patients with class C1. SARS-CoV-2 infection often resulted in the development of persistent systemic inflammation with elevated levels of cytokines and chemokines, including IFN-β, IFN-λ1, IFN-γ, IL-2, IL-6, IL-17, CXCL8, CXCL9, and CXCL10 [212]. Researchers reveal that Aβ1–42 peptide binds to the S1 subunit of the S protein and ACE2 in a SARS-CoV-2 pseudovirus model, facilitating viral entry and elevating IL-6 levels [17]. The S2 subunit of the S protein can enhance γ-secretase activity and increase Aβ production, as demonstrated in HeLa cells and infected mice brains [213].

Common features of AD and COVID-19 at the molecular pathway level included dysregulation of folate-mediated one-carbon metabolism, Cq10 deficiency, and oxidative stress due to both increased ROS production and damage to the antioxidant system, in particular glutathione synthesis. In both diseases, there is a general decrease in brain size and gray matter volume, as well as hypometabolism in the parahippocampal gyrus, thalamus, and cingulate gyrus. Endothelial dysfunction in COVID-19 and Alzheimer’s disease causes BBB disruption, activates microglia, and results in neuronal damage [20,212]. SARS-CoV-2 neuro-invasion was facilitated by NLRP3 inflammasome activation, which was similar to that described in the etiology of AD [17].

Bioinformatics and systems biology methods identified shared differentially expressed genes (DEGs) in brain tissue in relation to COVID-19 and neurodegenerative diseases (ND). Notably, 14 shared differentially expressed genes (DEGs) were identified in COVID-19 and AD. Among the hub genes, *CRH*, *SST*, *TAC1*, *SLC32A1*, *GAD2*, *GAD1*, *VIP*, and *SYP* were present. VIP helps protect against SARS-CoV-2 by reducing inflammation and viral replication in lung cells and preventing neurodegeneration caused by Aβ. *CRH*, *SST*, and *TAC1* are closely associated with neurotransmission and secretion in brain tissues, as well as with ND [34]. *SLC32A1* encodes the vesicular GABA transporter (VGAT), which is essential for GABA and glycine neurons [153]. Research indicates that GABAergic system components decline in the brain early in AD, prior to any changes in glutamatergic transmission [160,214].

Another study [33] attempting to explain the neurological consequences of SARS-CoV-2 infection analyzed genes that were co-expressed with *ACE2* and *TMPRSS2* genes. It was found that 93% of the 100 genes that correlated most closely with them had identical functions. They were grouped into several clusters: (1) ND and behavior; (2) immunity; (3) inflammation; (4) olfactory receptor; (5) cancer/apoptosis; and (6) executive function. The co-expressed genes were involved in several pathways, including the nicotinic pathway (dopaminergic neuron); TGF-β; CREB; ion channel transport; ERK, IL-2, and NF-kB signaling; PI3K/Akt; tyrosine kinases/adapters; INF-γ signaling; catabolism of His, Lys, Phe, Tyr, Pro, and Trp; AK metabolism; apoptosis; autophagy; the ATF-2 transcription factor network; and regulation of TP53 activity in relation to the DNA damage response (ATM-dependent only) [33].

COVID-19 resulted in the activation of pathways that are also active in AD. Protein secretion, cellular secretion, and receptor–ligand pathways were mainly impacted, along with inflammatory response pathways like cAMP, IL-17, MAPK, and PI3K-Akt [34]. The transcription factors FOXC1, GATA2, JUN, YY1, and RELA were also overexpressed [34]. SARS-CoV-2 infection might enhance Alzheimer’s symptoms by inhibiting mACE2, causing excessive inflammation, and decreasing BDNF production [17,215]. A study shows that recombinant Spike protein and toxin-like peptides in COVID-19 patients might damage brain function in human neural stem cell cultures [32]. Without affecting cell viability, they altered the expression of genes that are involved in NSC self-renewal/proliferation (*PAX6*) and neuronal and glial differentiation. Several genes showed notable changes in expression: *SPHK1*, *ELN*, *HEY1*, and *GASK1B* increased significantly due to peptide influence, while *UTS2* decreased. *SPHK1* encodes sphingosine kinase 1, which is associated with the control of cell survival, migration, and inflammation, and regulates microglial phagocytosis. Deregulation of *SPHK1* expression has been observed in AD, among others. *ELN* encodes elastin, which plays a neuroprotective role in ischemia–hypoxia brain injury. *HEY1* serves as a crucial effector of Notch3, and its overexpression in mouse neural progenitor cells encouraged astrocyte differentiation and reduced neuronal differentiation. *GASK1B* encodes Golgi-associated kinase 1B, which is involved in caveolae biogenesis, among other things [32]. Caveolae are involved in signal transmission from external ligands to internal pathways and play a role in cholesterol metabolism. The proteins required for their formation, caveolins, are expressed in many cell types. Caveolin-1 is found in hippocampal neurons, and its levels increase in response to Glu stimulation, depending on the dose. This occurs via kainate and AMPA receptors, leading to higher intracellular Ca^2+^ levels. Increased intracellular Ca^2+^ leads to the activation of CREB via CaMKII. Mild excitotoxicity results in increased caveolin expression, which may affect a variety of biochemical pathways [216]. Caveolin-1 protein and mRNA levels were almost twice as high in the hippocampus and frontal cortex of AD patients as in controls, suggesting that cholesterol regulation in brain cells is disrupted [217]. *UTS2*, which encodes urotensin-II, is associated with *ACE2* and *TMPRSS2* in brain regions like the hypothalamus, insula, amygdala, medulla oblongata, and pons’ parabrachial nuclei. Compared with Spike protein, peptides had a more pronounced negative effect on *UTS2* and *ACE2* expression [32].

Spike protein and peptides slightly dysregulated genes that are linked to glutamatergic (*GAP43*, *GRIA1*, *GRIA2*, *GRIA3*), GABAergic (*GABRA3*), dopaminergic (*NR4A2*, *TH*), and cholinergic (*CHAT*, *SLC5A7*, *SLC18A3*) systems. Abnormal *OLIG1* and *MBP* gene expression indicates oligodendrocyte development issues, whereas high *GFAP* expression suggests astrogliosis and microglial activation [32].

During the pandemic, decreased circulating Gln levels were found in several groups of COVID-19 patients, which correlated with disease severity. A decreased ratio of Gln to Glu suggests that patients are utilizing Gln more extensively [150]. Since ACE2 is expressed in both glutamatergic and GABAergic neurons, SARS-CoV-2 infection could initiate and/or exacerbate an imbalance between excitatory and inhibitory neuronal circuits, leading to excitotoxicity and cell loss, which also occurs during AD progression [17].

## 8. Concluding Remarks and Future Prospects

**Figure 4:**
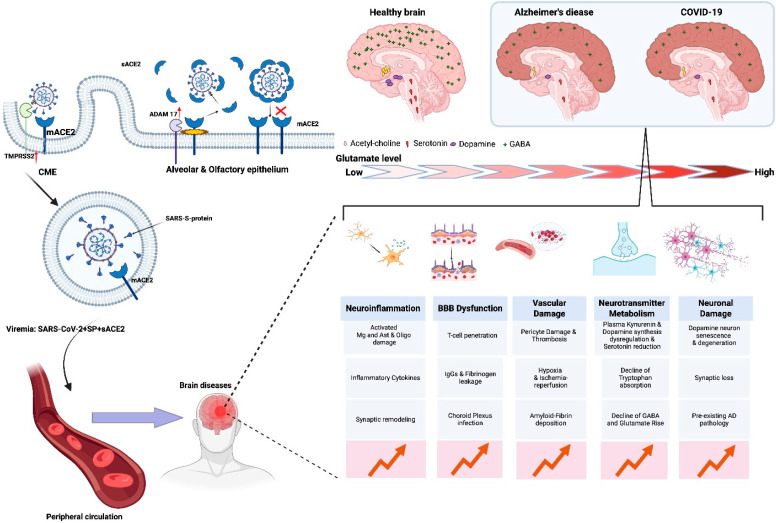
SARS-CoV-2 infection may cause damage in neurotransmitter systems in a similar manner to Alzheimer’s disease. The marked decline in the enzymatic activity of mACE2, fueled by heightened ADAM17 and TMPRSS2 activation, together with SARS-CoV-2, could aggravate RAS imbalance (Ang II/ACE2/Ang(1–7)) in patients suffering from particular comorbidities, including neurodegenerative (including Alzheimer’s/Parkinson’s diseases) and cardiovascular diseases, diabetes, and chronic lung illnesses. High CHI3L1, ADAM17, and TMPRSS2 activity is characteristic of these comorbidities. SARS-CoV-2 virions attach to host receptors, triggering clathrin-mediated endocytosis (CME), where proteases like TMPRSS2 process the S-protein (SP) and mACE2. This process helps the virus fuse with the mucus membranes of mainly the olfactory system (OS) and lungs. The virus can spread through the OS and bloodstream, allowing it to infect the choroid plexus and brain cells that carry ACE2, along with the proteases TMPRSS2, CTSL, and FURIN. Virus attachment also triggers a change in the cytoplasmic ACE2 tail that enhances ADAM17 activation. When ADAM17 is activated, it releases sACE2, which can either prevent or enhance virus entry through the ATR1 receptor. Unexpectedly, the cytoplasmic ACE2 tail, as well as ADAM17 expression, proved to be necessary for SARS-CoV infection. TMPRSS2 and ADAM17 work together to cut ACE2’s tail, which protects lung and heart tissues from RAS activation. Nonetheless, TMPRSS2 alone cannot yield enzymatically active sACE2. Hence, TMPRSS2-mediated ACE2 cleavage would be more harmful to the host. Increased levels of TMPRSS2 from androgen hormones and/or CHI3L1 can affect ACE2 cleavage, decrease mACE2 and sACE2, and impair RAS function. This hypothesis implies that the increased TMPRSS2 in the nasal epithelial cells of Black individuals might explain why they face higher rates of COVID-19 infection and poorer outcomes through enhanced cleavage of SARS-CoV-2 and ACE2. This figure was designed using an image from BioRender (https://www.biorender.com) [8,18,27,76,96,155,161,187,189].

During the COVID-19 pandemic, studies showed changes in gene expression and metabolism that could trigger the development of AD. Recent studies back these concerns, highlighting genes that are related to proteins like ACE2, BDNF, APOE4, and GAD. This review summarizes how changes in ACE2’s expression, isoforms, and interactions with other proteins in the brain due to SARS-CoV-2 infection may influence the development of neurodegenerative processes that are akin to Alzheimer’s disease. ACE2 performs its functions in a cellular- and tissue-specific manner. This occurs through enzymatic activity in the Ang (1–7)/MasR axis of the RAS, degradation of substrates in the kallikrein–kinin system, and regulation of non-catalytic activities such as transport and adhesion. The literature suggests that ACE2 mainly shows enzymatic activity in the brain, depending on its cellular location, but it likely also has non-catalytic functions. Changes in mACE2 levels, influenced by the Spike protein or SARS-CoV-2 infection, can disrupt various pathways and trigger neurodegenerative processes in the brain in a similar manner to Alzheimer’s disease (Figure 4). We focused on the lesser-known role of ACE2 in the regulation of neurotransmitter systems, which is crucial in many neurodegenerative diseases. This contrasts with other widely discussed topics like neuroinflammation, blood–brain barrier disruption, and oxidative stress [218].

As stated previously, it is apparently not the virus itself but the imbalance in the activity of the two branches of the RAS caused by it that may have been one of the main mechanisms of the progression of COVID-19 complications [97]. The mechanisms that govern this delicate balance remain inadequately explored. Studying how the Spike protein influences the ACE2-related RAS and neurotransmitter system is crucial, as they may significantly contribute to severe cognitive issues. It is important to address the many unanswered questions regarding the medium- and long-term effects of Spikeopathy on the nervous system. The findings underscore the importance of observing long-term neurological effects to identify people who are at higher risk of serious side effects.

Despite men being more vulnerable to COVID-19, experiencing higher mortality rates [219], women exhibited a greater prevalence of long-COVID neurological symptoms [220]. Studies have shown that patients with neurological long-COVID have changes in the brain areas responsible for cognitive functions [221]. Apparently, the central nervous system of women was more sensitive to the effects of the virus. This phenomenon is primarily linked to the polymorphism of the *ACE2* gene [222] and the intricate regulation of *ACE2* gene expression, which is situated on the X chromosome [223]. This regulation is influenced by sex hormones, as well as gender-specific immune responses that vary with age [224]. The sex-specific innate immune responses to viral infections, as well as vulnerability to neurodegenerative diseases, notably AD [225,226,227], is possible lead to more risk of developing AD after COVID-19 in older women.

Is there any light in the long, dark corridor? Recent research underscores the urgent necessity for careful consideration when directly targeting ACE2 through genetic approaches or pharmaceutical interventions [228,229]. This includes strategies advocating for personalized Alzheimer’s disease treatments based on identified neurotransmitter and ion modulations [230], as well as examining the influence of APOE4 on the development of both Alzheimer’s and SARS-CoV-2 [231], alongside neuroimaging biomarkers that were identified in individuals who have recovered from COVID-19 [232]. GABA and GABA agonists, along with lifestyle changes such as physical exercise and antioxidant/nutritional supplements, could help with AD prevention [16,233]. Correcting the RAS imbalance by enhancing ACE2 DIZE expression [234] or using AT1R blockers also appears promising for the prevention of AD [235,236]. New research reveals that COVID-19 impacts neuroimmune and glial functions in Alzheimer’s patients, emphasizing the importance of communication between the peripheral and central neuroimmune systems in neurodegenerative diseases [237,238]. Innovative therapeutic approaches focused on CHI3L1, including small-molecule inhibitors and neutralizing antibodies, have displayed significant potential in preclinical research, effectively diminishing neuroinflammation, lowering amyloid plaque buildup, and enhancing neuronal survivability, along with neural stem cell recovery [77,239,240,241]. Despite the therapeutic promise associated with targeting CHI3L1, the development of safe and precise treatments is fraught with challenges, especially regarding the ability to traverse the blood–brain barrier while minimizing side effects.

This review does not address all the molecular mechanisms related to ACE2, the damage caused by which during COVID-19 can trigger AD due to the complex nature of AD and limited knowledge about ACE2. Nevertheless, recent intensive studies on ACE2 have improved our understanding of this important protein and its functions in the body significantly. These investigations have shed light on how ACE2 interacts with biological processes and its role in health and disease. As a result, we now comprehend its importance more than ever before. This growing body of knowledge continues to shape future research directions and therapeutic strategies.

## Data Availability

No new data were created or analyzed in this study. Data sharing is not applicable to this article.
